# The miR-26a/AP-2α/Nanog signaling axis mediates stem cell self-renewal and temozolomide resistance in glioma

**DOI:** 10.7150/thno.33800

**Published:** 2019-07-28

**Authors:** Wenhuan Huang, Zhe Zhong, Chang Luo, Yuzhong Xiao, Limin Li, Xing Zhang, Liu Yang, Kai Xiao, Yichong Ning, Li Chen, Qing Liu, Xiang Hu, Jian Zhang, Xiaofeng Ding, Shuanglin Xiang

**Affiliations:** 1 State Key Laboratory of Developmental Biology of Freshwater Fish, College of Life Science, Hunan Normal University, Changsha, 410081, China;; 2 Key Laboratory of Protein Chemistry and Development Biology of State Education Ministry of China, College of Life Science, Hunan Normal University, Changsha, Hunan, 410081, China;; 3 The National & Local Joint Engineering Laboratory of Animal Peptide Drug Development, College of Life Science, Hunan Normal University, Changsha, 410081, China;; 4 Department of Neurosurgery, Hunan Provincial Tumor Hospital, The Affiliated Tumor Hospital of Xiangya Medical School of Central South University, Changsha, Hunan, 410013, China;; 5 Aier School of Ophthalmology, Central South University; Aier Eye Institute, Changsha, Hunan, 410015, China;; 6 Department of Endocrinology, Endocrinology Research Center, Xiangya Hospital of Central South University, Changsha, Hunan 410008, China;; 7 College of Engineering and Design, Hunan Normal University, Changsha, Human, 410081, China;; 8 Department of Biochemistry and Molecular Biology, University of New Mexico Health Sciences Center, Albuquerque, NM, 87131, USA;; 9 Department of Neurosurgery, Xiangya Hospital of Central South University, Changsha, Hunan, 410008, China.

**Keywords:** AP-2α, glioma, glioblastoma stem cells (GSCs), TMZ resistance, Nanog, STAT3, miR-26a

## Abstract

Aberrant expression of transcription factor AP-2α has been functionally associated with various cancers, but its clinical significance and molecular mechanisms in human glioma are largely elusive.

**Methods:** AP-2α expression was analyzed in human glioma tissues by immunohistochemistry (IHC) and in glioma cell lines by Western blot. The effects of AP-2α on glioma cell proliferation, migration, invasion and tumor formation were evaluated by the 3-(4,5-dimethyNCthiazol-2-yl)-25-diphenyltetrazolium bromide (MTT) and transwell assays* in vitro* and in nude mouse models *in vivo*. The influence of AP-2α on glioma cell stemness was analyzed by sphere-formation, self-renewal and limiting dilution assays *in vitro* and in intracranial mouse models *in vivo*. The effects of AP-2α on temozolomide (TMZ) resistance were detected by the MTT assay, cell apoptosis, real-time PCR analysis, western blotting and mouse experiments. The correlation between AP-2α expression and the expression of miR-26a, Nanog was determined by luciferase reporter assays, electrophoretic mobility shift assay (EMSA) and expression analysis.

**Results:** AP-2α expression was downregulated in 58.5% of glioma tissues and in 4 glioma cell lines. AP-2α overexpression not only reduced the proliferation, migration and invasion of glioma cell lines but also suppressed the sphere-formation and self-renewal abilities of glioma stem cells *in vitro*. Moreover, AP-2α overexpression inhibited subcutaneous and intracranial xenograft tumor growth *in vivo*. Furthermore, AP-2α enhanced the sensitivity of glioma cells to TMZ. Finally, AP-2α directly bound to the regulatory region of the Nanog gene, reduced Nanog, Sox2 and CD133 expression. Meanwhile, AP-2α indirectly downregulated Nanog expression by inhibiting the interleukin 6/janus kinase 2/signal transducer and activator of transcription 3 (IL6/JAK2/STAT3) signaling pathway, consequently decreasing O6-methylguanine methyltransferase (MGMT) and programmed death-ligand 1 (PD-L1) expression. In addition, miR-26a decreased AP-2α expression by binding to the 3' untranslated region (UTR) of AP-2α and reversed the tumor suppressive role of AP-2α in glioma, which was rescued by a miR-26a inhibitor. TMZ and the miR-26a inhibitor synergistically suppressed intracranial GSC growth.

**Conclusion:** These results suggest that AP-2α reduces the stemness and TMZ resistance of glioma by inhibiting the Nanog/Sox2/CD133 axis and IL6/STAT3 signaling pathways. Therefore, AP-2α and miR-26a inhibition might represent a new target for developing new therapeutic strategies in TMZ resistance and recurrent glioma patients.

## Introduction

Gliomas are the most aggressive, common and devastating primary brain tumors, and have a dismal prognosis and limited treatment options 1. The standard of care for glioma patients includes surgery followed by combined radiation and chemotherapy. TMZ is a first-line treatment for glioblastoma as well as other tumors that prolongs overall survival, with a median survival of approximately 15 months and a 5-year survival rate of less than 10% 2. However, TMZ is only beneficial to a subgroup of patients lacking MGMT 3. Recurrence after standard therapy is inevitable, ultimately resulting in a high mortality for glioma patients, and is the most challenging reality facing doctors and patients. Tumor initiation, therapeutic resistance and recurrence originate from glioma stem cells (GSCs) 4, 5. Elucidation of the molecular pathways in GSCs may be essential for understanding glioma stemness and TMZ resistance. Therefore, the identification of novel targets and insight into molecular events are urgently needed for the development of more effective therapeutic strategies for glioma patients.

The transcription factor AP-2α, first identified as a fundamental regulator of mammalian craniofacial development 6, 7, has been closely associated with various tumor malignancies 8-10. Exogenous AP-2α inhibited the proliferation and growth of cancer cell lines *in vitro* 8, and reduced the tumorigenicity and metastatic potential in the nude mice* in vivo*, whereas inactivation of AP-2α reversed the tumor inhibitory effects 9, 11. Some data showed that a high proportion of AP-2α nucleoplasm localization was related to increased tumor malignancy and poor survival in certain tumor subtypes 12-14. The expression of AP-2α together with p21 was correlated with recurrence-free survival in colorectal carcinoma patients 15. Conversely, AP-2α activated Hoxa7/9 and the Hox cofactor Meis1 to enhance the proliferation and cell survival of acute myeloid leukemia (AML) cells 16. Thus, AP-2α acts as a bifunctional transcription factor in the development and progression of carcinogenesis by influencing multiple signaling pathways, including VEGF, PI3K/AKT, Wnt/β-catenin, Apaf1/caspase 9, HIF and p53, to modulate angiogenesis, cell proliferation, invasion and the microenvironment 11, 17-21. Importantly, AP-2α suppressed the sphere formation and renewal abilities of hepatocellular carcinoma stem cells 22. AP-2α expression sensitized cancer cells to chemotherapy drugs and enhanced tumor killing, while AP-2α deletion led to drug resistance 23-25, suggesting the significance of AP-2α in tumor recurrence and clinical therapies.

Although AP-2α expression negatively correlates with the human glioma grade 26, the molecular mechanisms of AP-2α in glioma are not clearly elucidated. In the present study, we showed AP-2α downregulation in human glioma cells. Moreover, the tumor suppressive effect of AP-2α was exerted through inhibiting the transcriptional activity of the Nanog gene and the IL6/STAT3 signaling pathway to attenuate the stemness and TMZ resistance of glioma cells. In addition, oncogenic miR-26a bound to the 3' UTR of AP-2α to decrease its expression and reversed its effects on glioma. Therefore, AP-2α markedly suppresses the proliferation, stemness and TMZ resistance of glioma cells, suggesting that it could serve as a novel therapeutic target to evaluate the prognosis of primary and recurrent glioma patients. miR-26a inhibition combined with TMZ might offer effective therapy strategies for glioma patients.

## Methods

### Cell culture

Human glioma cell lines U251, U87, A172, SHG44, human cervical cancer cell line HeLa, human Embryonic Kidney 293 and 293T cells were purchased from the American Type Culture Collection (ATCC). All cells were cultured in Dulbecco's modified Eagle's medium (DMEM, Thermo Scientific, Waltham, MA, USA) supplemented with 10% fetal bovine serum (FBS, Thermo Scientific, Waltham, MA, USA), 100 IU/mL penicillin G and 100 μg/mL streptomycin (Invitrogen Life Technologies, Carlsbad, CA, USA). Cells were maintained in a humidified atmosphere containing 5% CO_2_ at 37 ˚C.

### Immunoblotting (IB), Immunohistochemistry (IHC) and Immunofluorescence (IF)

For immunoblotting, cultured cells were harvested, washed with phosphate buffered saline (PBS), and lysed in radioimmunoprecipitation assay (RIPA) buffer as described previously 27. Antibodies used are listed as following, mouse monoclonal antibodies against AP-2α (sc-12726), cyclin D1 (CCND1) (sc-4074), c-Myc (sc-4084), β-actin (sc-58673) and GAPDH (sc-47724) were from Santa Cruz Biotechnology (Santa Cruz, CA). Rabbit polyclonal antibodies against STAT3 (12640) and phosphorylated STAT3 (Y705) (9145), Bcl-2 (4223), Pro-caspase3 (14220) were from Cell Signaling Technology (MA, USA). Rabbit polyclonal antibodies against JAK2 (A7694), phosphorylated JAK2 (Y1007/1008) (AP0531) and IL-6 (A2447) were from AbClonal Technology (MA, USA). Rabbit polyclonal antibodies against Nanog (D262945), Sox2 (D164316) and GFAP (D162817) were from Sangon Biotech (Shanghai, China). Rabbit polyclonal antibody to CD133 (BA3141) was from Boster Biological Technology (CA, USA). HRP-conjugated goat anti-rabbit (A6667) and goat anti-mouse (A5278) secondary antibodies were from Sigma (St. Louis, MO, USA). The signal was detected with SuperSignal West Pico chemiluminescent Substrate (Thermo Scientific Pierce, Rockford, IL, USA) and visualized with tanon-5200 system (Bio-Tanon, Shanghai, China).

For immunohistochemical (IHC) analysis, human glioma tissues and normal brain tissues were examined (Table 1). These experiments were approved by Human Ethics Committee of Hunan Normal University and informed consent was obtained from all patients. The IHC analysis was performed on polyformalin-fixed and paraffin-embedded tissues as previously described 22, 28. Tissue sections were incubated with indicated primary antibodies against AP-2α (3B5) (A0416) (1:200), Nanog (AF5388) (1:200), Sox2 (A0561) (1:200), CD133 (A0219) (1:200), p-STAT3 (AF3293) (1:100, AbClonal Technology) and Ki67 (9449) (1:200, cell signaling technologies) or normal mouse IgG control (sc-2025) (1:200, Santa Cruz, CA). The percentage of tumor cells stained was scored as 0 (no cell staining), 1 (≤30%), 2 (31-60%) and 3 (61-100%). Staining between two score values was given 0.5.

For IF staining, glioma cells were cultured on glass coverslips in a 12-well plate and grown to 70% confluence, cells were treated as described previously 29, 30. The primary antibodies used were mouse monoclonal antibodies against AP-2α (sc-12726) (Santa Cruz, CA), rabbit polyclonal antibodies against Nanog (AF5388), CD133 (BA3141) and Nestin (BA1289) (Boster Biological Technology) while the secondary antibodies were Alexa Fluor 488 phalloidin (A12379) and 594 dye (A12381) (Invitrogen), the nucleus were visualized by Hoechst 33258 staining (14530) (Sigma). The fluorescent signals were examined using an upright fluorescence microscope (Zeiss Axioskop 2).

### Generation of stable expression cell lines using lentivirus

Lentiviral particles were prepared as described in our previous work 22. Briefly, the lentivirus AP-2α overexpression plasmid and packaging plasmids (pHelper 1.0 and pHelper 2.0) were cotransfected into 293T cells, supernatants were harvested 48 h after transfection and filtered through a 0.45-μm pore size filter (Millipore, Billerica, MA, USA) and concentrated by ultracentrifugation. The infectious titer was determined using hole-by-dilution titer assay. Glioma cells were infected with AP-2α-lentivirus or NC-lentivirus at the multiplicity of infection (MOI) of 1 in the presence of 5 μg/mL polybrene (Sigma) and detected on the 4th day by the invert fluorescence microscope followed by resistance screening of 1.5 μg/mL of puromycin for stable cell lines.

The sequence of pre-miR-26a was cloned to pLenti-GFP lentiviral vector pEZX-MR03 (GeneCopoeia) and the inhibitor (single-stranded complementary sequence to the mature miR-26a) of miR-26a was inserted to lentiviral vector H1-MCS-CMV-EGFP (GeneCopoeia). Recombinant and control vectors were then transfected into HEK293T cells with the Lenti-Pac HIV Packaging Mix (GeneCopoeia), and viral supernatants were collected and purified 48 h after transfection. Stable miR-26a-infected and miR-26a-inhibitor-infected glioma cells were screened by 1.5 μg/mL of puromycin 96 h after infection.

### Cell proliferation assays

For cell survival assays, 100,000 cells stably expressing AP-2α or NC were plated in triplicate in 6-well plates in complete DMEM medium. After 1-5 days, cell numbers were counted with a hemocytometer. To detect the cell growth rate, 5000 cells per well were cultured in 48-well plates. From day 1 to 7, cells were incubated with MTT reagent (Sigma) for 4 h at 37 ˚C. Then 100 μL dimethyl sulfoxide (DMSO) per well was added to dissolve the formazan crystals. The absorbency at 490 nm was measured with a spectrophotometer (UV-2102C, Changsha, China). For TMZ treatment, glioma cells were seeded at 3000 cells/well in 96-well plates treated with increasing concentrations of TMZ (10-400 µm) for 48 h followed by MTT assays. At least three independent assays were performed in octuplicate.

### Cell migration and invasion assays

The transwell cell migration and invasion assays were performed in polyethylene terephthalate (PET)-based migration chambers and BD BioCoat Matrigel Invasion Chambers (BD Biosciences, Bedford, MA, USA) with 8 μm porosity as described previously 31. The same number of tumor cells (2×10^4^) in serum-free DMEM were seeded onto uncoated or Matrigel-coated filters in the upper chambers. DMEM/F12 (Gibco-Invitrogen, Carlsbad, CA, USA) with 15% FBS was added to the lower chambers. After 36-48 h of incubation, cells on the upper surface of the filters were removed with a cotton swab, and the filters were fixed with 100% methanol and stained with crystal violet. The migration and invasive ability of glioma cells was calculated as the mean number of cells in all fields and presented as the proportion of migrated and invaded cells relative to initial cells. The experiments were carried out three times, individually. 

### Sphere-forming, self-renewal, CD133 positive cell sorting and limiting dilution assays

Suspensions of single-cells were seeded into 6-well plates at a density of 5,000 cells/mL in stem cell-conditioned medium containing DMEM/F12 supplemented with 100 IU/mL penicillin G, 100 μg/mL streptomycin, 10 ng/mL EGF (Peprotech Inc., Rocky Hill, NJ, USA), 10 ng/mL bFGF (Peprotech Inc., Rocky Hill, NJ, USA), and 1×B27 (Invitrogen). The CD133 positive cells were separated by magnetic cell sorting technique (MACS). The sorting process was performed according to the instruction of CD133 cell isolation kit from Miltenyi Biotec GmbH (Bergisch Gladbach, Germany). Culture suspensions were passaged every seven days when spheroid diameters were at least 50 µm taken by photography. The sphere-forming cells were analyzed by counting the number of cells and spheres on day 10.

To investigate self-renewal capacity of glioma stem cells (GSCs), primary gliospheres were incubated with Accutase, dissociated into single cells and replated in 96-well plates with 200 μL/well of stem cell-conditioned medium. The number of spheres were determined after 7 days. The* in vitro* limiting dilution assay (LDA) was performed as described previously 32. Briefly, GSCs cultured were collected, dissociated into single cells and seeded in 96-well plates at a density of 5, 10, 20, 50, 100 or 200 cells per well and each well was then examined for formation of tumor spheres after 9 days. Wells without tumor spheres were counted for each group.

### *In vivo* functional assays

The mouse experiments were performed according to the ethical guidelines for laboratory animal use and approved by the Ethics Committee of Hunan Normal University. For subcutaneous tumor models, approximately 2×10^7^ of lentivirus-infected U87 cells in 0.2 mL of sterile PBS were injected subcutaneously into the left and right dorsal regions of 4-week-old female nude mice (n=6 mice/group), respectively. Mice were checked every 2 days. After 25 days, mice were sacrificed, tumors were excised, weighed and photographed. The formed tumors were measured and analyzed by Hematoxylin and Eosin (H&E) staining and IHC analysis as described previously 33.

For intracranial xenograft tumor models, female nude mice (n=6 mice/group) at 6 weeks of age were anesthetized and placed into stereotactic apparatus equipped with a z axis (Stoelting Co, Chicago, IL, USA). A small hole was bored in the skull 0.5 mm right to the midline and 2.0 mm posterior to the bregma using a dental drill as described previously 34. Stem cells (3×10^5^) in 3 μL PBS or glioma cells (5×10^5^) in 5 μL PBS were injected into the right caudate nucleus 3 mm below dura mater of the brain over a 3 min period using a 5 μL Hamilton syringe with fixed needle. If the drug was used, one week post injection, mice were treated with TMZ at a concentration of 25 mg/kg body weight by intraperitoneal injection every other day for 2 weeks. Mice with neurological deficits or moribund appearance were sacrificed. Brains were fixed using transcranial perfusion with 4% paraformaldehyde (PFA) and post-fixed by immersion in 4% PFA for paraffin embedded tissues, then analyzed by conventional Hematoxylin and Eosin (HE) and IHC staining.

### Flow cytometry analysis

Glioma cells and gliospheres were incubated with Accutase and repeatedly pipetted with a pipette to disperse the spheres into a single state, and washed twice with cold PBS. The cells were centrifuged at 500× g for 5 min and resuspended in binding buffer, then Annexin V-FITC (88-8005-72) and propidium iodide (PI) (00-6990-50) or CD133-FITC antibody (11-1339-42) (eBioscience, invitrogen) and anti-IgG FITC (31531) (invitrogen) were added and incubated in the dark at room temperature for 15 min. The samples were then analyzed by a FACSCalibur flow cytometer (BD Biosciences, CA, USA) and FlowJo software.

### RNA preparation, cDNA synthesis and real-time PCR

Total RNA was extracted from glioma cell lines and tissues using TRIzol reagent (Invitrogen, Carlsbad, CA, USA), and then reverse transcribed into cDNA using M-MLV RTase and random primer (GeneCopoeia, Guangzhou, China). SYBR green (Takara Bio Inc., Shiga, Japan)-based real-time PCR was performed using ABI 7900 thermocycler (Thermo Fisher Scientific, MA, USA) as described previously 31. The reactions were incubated in a 96-well plate at 95 °C for 10 min followed by 40 cycles of 95 °C for 15 s and 60 °C for 30 s. Quantitative PCR primers were shown in Table S1. The Ct value was measured during the exponential amplification phase. The relative expression levels of target genes were given by 2^-ΔΔCt^ and log_2_ values were presented as the relative changes compared to the controls.

### Luciferase reporter assays

The regulatory region and mutated sequences of the Nanog gene were cloned into pGL3-Basic vector (Promega Corporation, Madison, WI, USA). The wildtype and mutated AP-2α 3' UTR were inserted into plasmid pmirGLO (Promega) 23. The full-length STAT3 was cloned into the pCMV-Myc vector. HEK293 cells were cultured in 12-well plates and transfected with the indicated plasmids together with reporter plasmid using Lipofectamine 2000 as previously described 35. For luciferase assays, cells were cultured for 36 h after transfection or treated with 100 ng/mL of IL-6 for 8 h before harvest, and cell lysate was used to measure luciferase reporter gene expression using the luciferase reporter assay system (Promega). All experiments were performed in triplicate and repeated at least three times.

### Electrophoretic mobility shift assay (EMSA)

EMSA experiments were performed as described previously 29, 36. The recombinant GST-AP-2α protein was purified as described previously 30. The specific hot probes covering AP-2 binding sites in the Nanog regulatory region were synthesized and labeled with biotin at the 3' end. The sequences of specific probes were listed in Table S1. Following the manufacturer's instructions of the EMSA/Gel-Shift Kit (Beyotime, Shanghai, China), the binding reaction was carried out in a mixture containing 5 μg GST-AP-2α and 2 pmol biotin-labeled wild-type or mutated sequences in 10 μL of binding buffer with or without unlabeled (cold) probes pre-incubated for 30 min at room temperature. The reaction mixtures were loaded onto a 4% nondenatured gel at 100 V for 60 min and transferred onto nylon membrane to perform chromogenic reaction.

### Statistical analysis

Statistical analyses were performed using the SPSS 16.0 (SPSS Inc., Chicago, IL, USA) and GraphPad Prism software (SanDiego, California, USA). The Pearson′s χ2 test was used to analyze the association of gene expression and clinicopathologic characteristics. The expression levels of target genes in glioma tissues were compared using a paired Student's *t*-test. Differences between gene expression were assessed by Fisher′s exact test. Survival analyses were assessed by Kaplan-Meier plotter. Data are shown as mean ± SD from at least three independent experiments. Results were considered statistically significant when *P*<0.05.

## Results

### AP-2α is expressed at low levels in glioma tissues and cell lines

**Figure 1:**
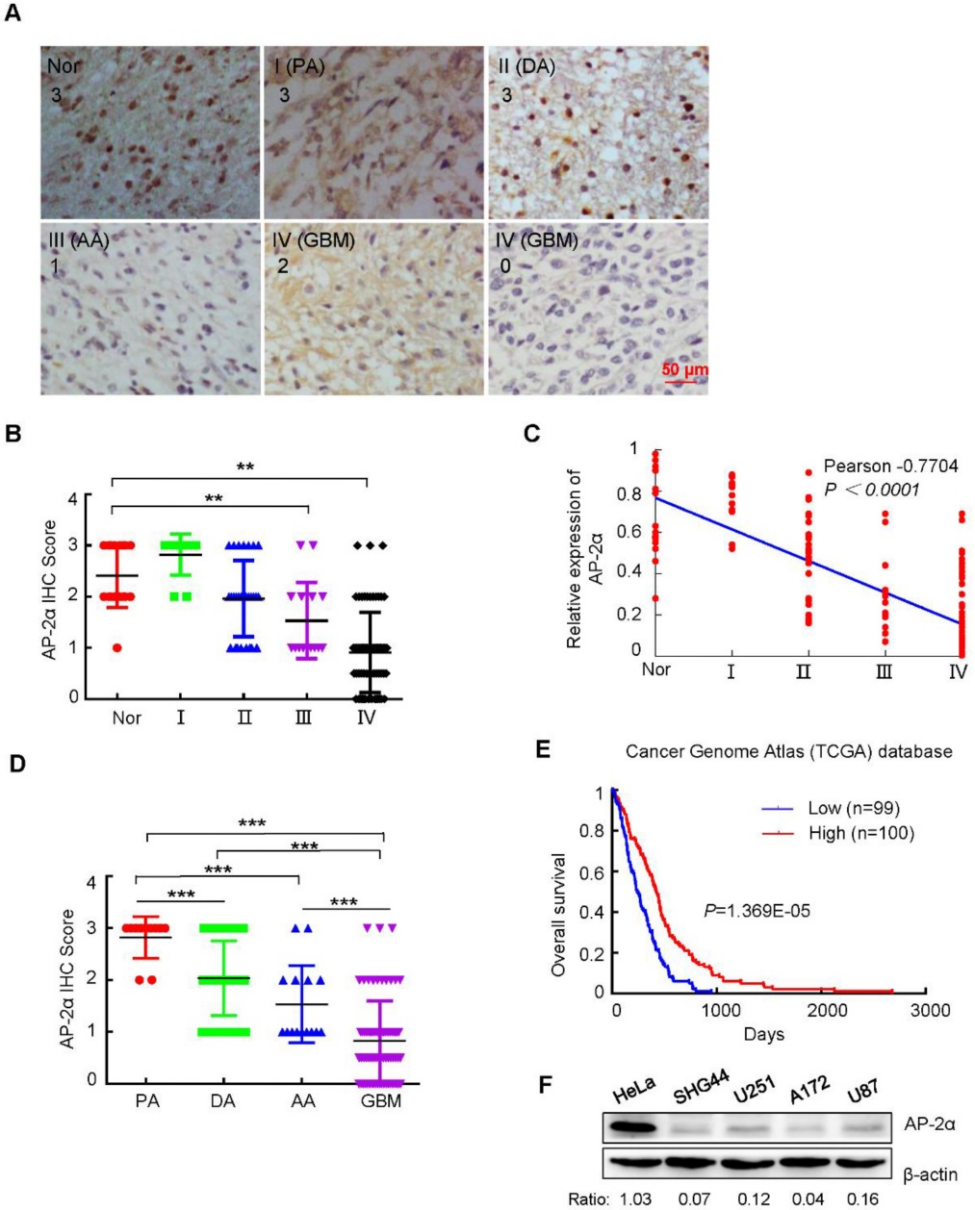
** The expression levels of AP-2α in glioma tissues and cell lines. (A)** AP-2α expression was examined by immunohistochemical analysis in 130 glioma tissues and 17 adjacent normal tissues. Strong nucleic expression of AP-2α (brown staining) was detected in normal tissues and stage I/II glioma cells, and the nucleus was stained blue with hematoxylin. The staining intensity was scored with grades 0-3. Nor, Normal; PA, pilocytic astrocytoma; DA, diffuse astrocytoma; AA, anaplastic astrocytoma; GBM, glioblastoma multiform. **(B)** Immunohistochemical scores of glioma and normal tissues stained with monoclonal anti-AP-2α antibody. Each symbol represents an individual sample. Statistical comparisons of AP-2α expression between glioma and normal tissues were performed according to the SPSS software. **, p<0.01. **(C)** The correlation of AP-2α expression and glioma grade was analyzed by Graphpad Prism. **(D)** Immunohistochemical scores of AP-2α expression in various histological types of glioma. ***, p<0.001. **(E)** The correlation of AP-2α expression and overall survival of glioma patients was determined from the Cancer Genome Atlas (TCGA) data. n: sample number. **(F)** AP-2α expression in glioma cell lines was detected by Western blotting. Relative AP-2α expression was quantified by Image J software using β-actin as an internal control.

To determine the clinical significance of AP-2α in glioma, the AP-2α expression in 11 WHO grade I, 24 WHO grade II, 14 WHO grade III and 81 WHO grade IV gliomas was examined by IHC analysis. AP-2α expression was mainly localized in the nucleus and detected in 21 (16.2%) of the 130 glioma tissue samples with strong staining (3+), in 34 (26.1%) glioma tissue samples with moderate staining (2+), and in 75 (57.7%) glioma samples with weak or negative staining (0~1+) (Figure 1A-B), which indicated that AP-2α was mostly expressed at low levels in glioma tissues (*P*<0.01). Moreover, AP-2α expression was significantly decreased in both grade III and IV glioma tissues compared with that in normal brain tissue and grade I-II glioma tissues (*P*<0.01*,* paired Student's *t*-test). Clinicopathological association analyses of the 130 glioma tissue samples revealed that AP-2α expression was significantly associated with age and histological diagnosis (Pearson's χ2 test*, P*<0.05; Table 1). In particular, AP-2α expression was negatively correlated with tumor grade (Pearson's correlation coefficient, -0.7704, *P*<0.0001; Figure 1C). Among the 81 glioblastoma 79% maintained AP-2α expression at a low level and among the 49 astrocytomas 22% had low expression of AP-2α (Figure 1D). Importantly, AP-2α was positively associated with overall patient survival based on data from The Cancer Genome Atlas (TCGA) (Figure 1E).

We then analyzed the protein expression of AP-2α in four glioma cell lines. Low expression or loss of AP-2α was evident in SHG44, U251, A172 and U87 cells compared to that in HeLa cells, which expressed AP-2α at a high level (Figure 1F). These results indicated that AP-2α is also expressed at low levels in glioma cell lines, in line with the observations in glioma tissues.

### AP-2α overexpression inhibits glioma progression *in vitro* and *in vivo*

**Figure 2:**
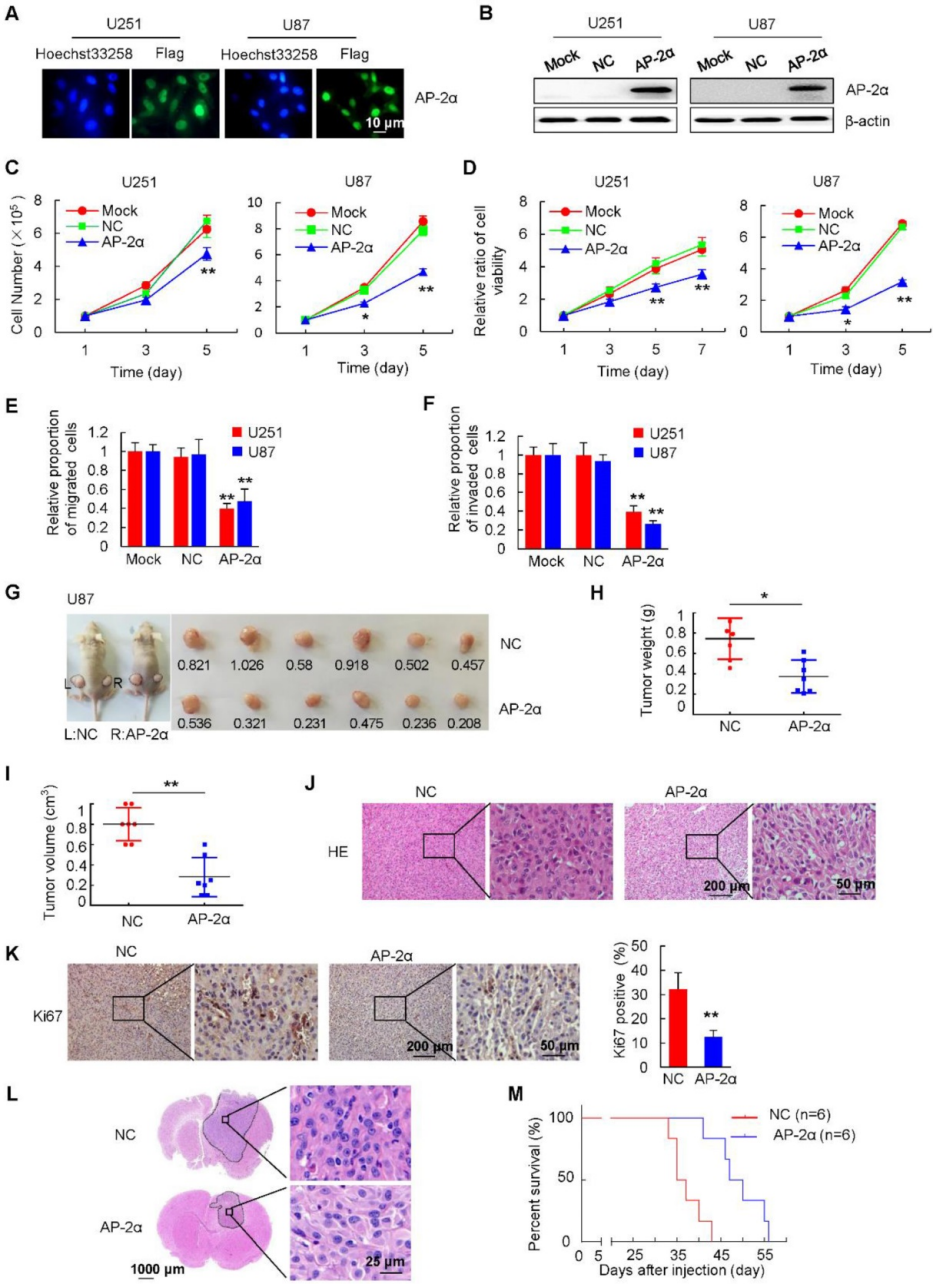
** Effects of AP-2α overexpression on glioma progression *in vitro* and* in vivo*. (A)** Immunofluorescence staining of Flag-AP-2α overexpression in lentiviral-infected glioma cells. **(B)** Flag-AP-2α expression in glioma cells was detected by Western blot using anti-Flag antibodies. β-actin was served as a loading control. **(C)** Cell survival assays of lentiviral-infected glioma cells and parental cells. Cells (100,000) were plated into 6-well plates in triplicate, grown in DMEM with 10% FBS for 1-5 days, cell numbers were counted with a hemocytometer. **(D)** MTT assays of lentiviral-infected glioma cells and parental cells. Cells (5,000) were plated in octuplicate in 48-well plates and grown in DMEM with 10% FBS. The absorbance at 490 nm was analyzed for 1, 3, 5 and 7 days. **(E)** Effect of AP-2α overexpression on cell migration as determined by transwell assays. Examples of cells migrated through the PET-membrane (pore size: 8 μm) and relative migration proportion of cells are shown. The proportion of migrated cells is based on total number of cells at the end of the assay relative to initial number of cells, which was set to 1 in the Mock group. **(F)** Effect of AP-2α overexpression on cell invasion through Matrigel matrix. Examples of cells migrated through Matrigel-coated transwell inserts and relative invasion proportion of cells are shown. The proportion of invaded cells is based on total number of cells at the end of the assay relative to initial number of cells, which was set to 1 in the Mock group. **(G)** AP-2α overexpression decreased the proliferation of U87 cells *in vivo*. About 2×10^7^ of lentivirus-infected cells were injected subcutaneously into the left and right back of female nude mice (BALb/c) (n =6 per group). After 25 days, tumors were excised, photographed, and measured. The weight and volume of the tumors excised **(H and I)** are mean ± SD in three independent experiments. **(J)** H&E staining was performed on serial sections of mouse tumors generated from glioma cells. **(K)** IHC analysis of Ki67 expression and quantification of Ki67-positive cells in mouse tumors are shown. **(L)** U87 cells (5×10^5^) were injected stereotactically into the brain of BALB/c nude mice using a 5 μL Hamilton syringe. Cell were injected in the middle of the craniotomy open window to a depth of 3 mm. H&E staining of brain tissues from the control and AP-2α overexpressing groups was shown. **(M)** Kaplan-Meier survival curves of nude mice bearing intracranial tumors of U87 cells in control group and AP-2α overexpressing group. n: mice number. These data represent at least three independent experiments with similar results. *, p<0.05, **, p<0.01, compared with controls. NC, negative control.

To further investigate the role of AP-2α in human glioma cells, AP-2α was cloned into the lentiviral vector pGC-FU-3Flag-IRES-Puromycin as described previously 22, and stable glioma cell lines overexpressing AP-2α (pFLAG-AP-2α) or a negative control (pFLAG-NC) were screened and established. The fluorescence intensity was markedly increased 4 days after infection, and the infection efficiency was over 98% in U251, U87 and A172 cells followed by puromycin selection (Figure 2A and Figure S1A). Western blot and Q-RT-PCR analyses demonstrated the overexpression of AP-2α in these glioma cell lines (Figure 2B and Figure S1B, S1C). Next, we examined whether AP-2α is a critical regulator of glioma cell proliferation and detected the effect of AP-2α overexpression on glioma cell growth. Glioma cells overexpressing AP-2α exhibited a decreased growth compared with that of negative control cells (Figure 2C and Figure S1D). Consistently, MTT assays showed that AP-2α overexpression resulted in a remarkable decrease in viable cells (Figure 2D and Figure S1E). The apoptosis rate of AP-2α overexpressing glioma cells was higher than that of control cells as determined by FACS analysis (Figure S1F). Transwell migration assays also showed that overexpression of AP-2α led to a marked decrease in cell migration, while the proportion of cells migrating through the 8-µm pores was higher in the control group than in the AP-2α group (Figure 2E and Figure S1G). Moreover, cell invasion assays revealed that AP-2α overexpression resulted in a significantly lower proportion of cell migrating through Matrigel-coated chamber compared with controls (Figure 2F and Figure S1H). These findings demonstrated that AP-2α overexpression inhibits glioma cell proliferation and cell motility *in vitro*.

To further examine the effect of AP-2α on the *in vivo* tumorigenicity of glioma cells, AP-2α-infected and NC-infected U87 cells were subcutaneously injected into the left and right dorsal flanks of nude mice, respectively. After 25 days, the average weight and volume of tumors in the AP-2α overexpression group were markedly reduced compared with those of tumors in the control group (Figure 2G-I). H&E staining confirmed that glioma cells were more loosely arranged in the AP-2α overexpression group than in the control group (Figure 2J). IHC analysis further confirmed that AP-2α overexpression inhibited the expression of Ki67, a proliferation marker in glioma (Figure 2K). We implanted AP-2α-infected and NC-infected U87 cells into the right frontal lobes of athymic mice (BALB/c) by intracranial injections. The mice were monitored daily for general appearance, behavioral changes, and neurological deficits. These mice were sacrificed when they became moribund. Mouse brain sections were stained with H&E to visualize and measure the tumor sizes. Strikingly, AP-2α (18.53 ± 17.9 mm^3^) reduced the intracranial tumor volume by about 75% compared with that in the NC group (73.79 ± 32.3 mm^3^) (*P*=0.04) (Figure 2L). AP-2α prolonged the survival of intracranial xenograft mice, as shown by Kaplan-Meier analysis (Figure 2M). The median survival time were 36 days for the control mice and 48.5 days for mice in the AP-2α group (*P*=0.0016). And AP-2α overexpression showed a longer tumor latency (8 days) than control cells. These results indicated that AP-2α overexpression markedly inhibits the tumorigenic ability of glioma cells.

### AP-2α overexpression attenuates gliosphere formation and expansion and inhibits the self-renewal and proliferation of GSCs and CD133^+^ glioma cells

**Figure 3:**
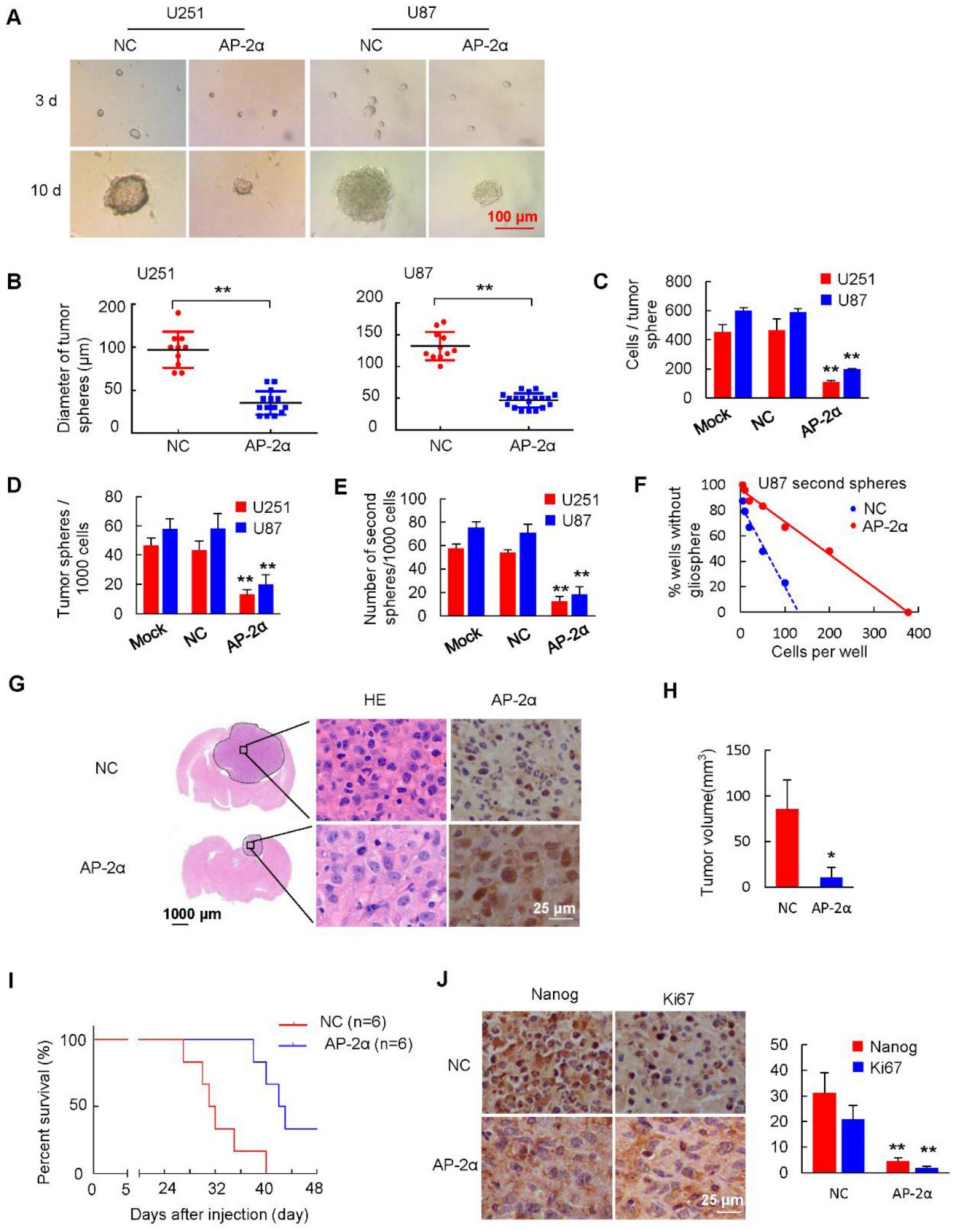
** AP-2α overexpression attenuates the self-renewal ability of GSCs. Representative images (A)** and diameter **(B)** of spheroid cells are shown. **(C)** Quantification of cell numbers per spheroid. Tumor spheres were collected and dissociated with trypsin for single cell suspension. **(D)** Evaluation of the number of spheres from 1000 cells. The number of primary spheres formed on day 9 is shown. **(E)** Evaluation of the number of secondary spheres from 1000 cells. Primary tumor spheres were dissociated, replated, and cultured. The number of secondary tumor spheres was quantified after 7 days. **(F)** Tumor sphere formation was measured through a limiting dilution assay. Cells from U87 secondary spheres were plated at 200, 100, 50, 20, 10, or 5 cells/well and cultured in stem cell-conditioned medium (n=48 wells/condition, *p* = 0.012). **(G)** U87 sphere cells (3×10^5^) were injected stereotactically into the brain of athymic mice (BALB/c nu/nu) using a 5 μL Hamilton syringe. H&E staining of brain tissues from the control and AP-2α overexpression groups and IHC analysis of AP-2α expression in mouse brain tissues were performed. **(H)** The volume of the intracranial tumors was measured and indicated as mean ± SD. **(I)** Kaplan-Meier survival curves of nude mice bearing intracranial tumors of U87 sphere cells in control group and AP-2α overexpression group. n: sample number. **(J)** IHC analysis of Nanog and Ki67 expression in mouse brain tissues was performed. All data are presented as the mean ± SD of three independent experiments. *, *P* < 0.05, **, p<0.01, compared with the NC group.

GSCs play a key role in tumor resistance to conventional therapies and recurrent diseases. GSCs are capable of self-renewal, differentiation and tumor formation 37. To investigate whether AP-2α functions in the characterization and differentiation of GSCs, we first detected the effect of AP-2α expression on gliosphere formation. Spheroids were visible on day 3 and increased in size and number between days 7 and10. AP-2α overexpression resulted in a visible decrease in the size of gliospheres compared with those in the NC group (Figure 3A-B). Among the control GSCs, 454 ± 49 U251 cells and 598 ± 21 U87 cells formed spheres, whereas the number of sphere-forming cells was significantly decreased (U251 109 ± 9, U87 194 ± 6, *P* <0.01) in the AP-2α-overexpressing GSC group (Figure 3C). Moreover, a single cell suspension was plated at a density of 1000 cells/well, and the formed spheres were counted after 10 days. GSCs overexpressing AP-2α showed significantly fewer gliospheres than the control U87 and U251 cells (Figure 3D). The U251 gliospheres retained the expression of the neural stem cell marker CD133 and nestin (Figure S2A).

To further detect the self-renewal capacity of tumor spheres, primary tumor-spheres were dissociated into single cells and replated at a density of 1000 cells/well in stem cell-conditioned medium. The mean number of secondary tumor spheres formed by AP-2α-overexpressing GSCs was lower than that of control U87 and U251 stem cells (Figure 3E). Next, we performed a limiting dilution assay to analyze secondary tumor-sphere formation in U87 cells at serial cell concentrations ranging from 200 to 5 cells/well. Consistently, all the dissociated primary U87 gliospheres exhibited the self-renewing ability to form secondary tumor spheres. However, AP-2α was found to generate a small number of secondary tumor spheres, while control gliosphere cells formed many spheres (Figure 3F and S2B). Moreover, the number of cells required to generate at least 1 tumor sphere/well was determined to be 130.90 in control gliospheres and 377 in AP-2α-overexpressing spheres. To further evaluate the proliferative and migratory abilities of disaggregated U87 gliosphere cells, MTT and stem cell survival assays were performed with gliosphere cell populations. AP-2α impaired the proliferative and survival capacities of GSCs compared with those of control cells (Figure S2C-D). In addition, AP-2α attenuated the migration and invasion capacities of GSCs compared with those of controls (Figure S2E-F). Taken together, our results suggest that AP-2α suppresses the self-renewal, proliferation and migration of GSCs.

Because such gliosphere cultures contain a heterogeneous mixture of cell types, CD133 is considered the critical marker denoting stem cell-like subpopulations 38. We next examined whether AP-2α overexpression influences the percentage of CD133^+^ glioma cells. As shown in Figure S3A, flow cytometric quantification of CD133 expression in glioma cells dropped from 34.0% in control cells to 5.64% in AP-2α-overexpressing glioma cells. Moreover, the percentage of CD133-positive cells among gliosphere cells (98.4%) was increased by ∼3-fold compared with that in glioma cells (34.0%), and AP-2α overexpression decreased the number of CD133-positive cells in gliospheres (Figure S3B). We also sorted CD133-positive cell populations from stable cell lines using magnetic bead cell sorting, sorted CD133^+^ cell populations had approximately 96.9% purity (Figure S3C). CD133^+^ glioma cells formed and expanded the gliospheres. However, AP-2α-overexpressing CD133^+^ populations suppressed sphere formation (Figure S3D). The limiting dilutions and cell counting assays showed that AP-2α inhibited the tumor self-renewal and proliferative capacity of CD133^+^ glioma cells (Figure S3E-G).

To further detect the inhibitory effects of AP-2α on GSC growth* in vivo*, U87 stem cells were intracranially injected into the right frontal lobe of BALB/c mice as described above. We found that AP-2α (10.96 ± 10.46 mm^3^) reduced the intracranial tumor volume by over 87% compared with that of tumors generated from NC stem cells (84.56 ± 26.78 mm^3^) (*P*=0.04) (Figure 3G-H). Intriguingly, AP-2α prolonged the survival of intracranial xenograft mice, as shown by the Kaplan-Meier anlysis (Figure 3I). The median survival of the control group mice was 31.5 days. In contrast, AP-2α increased the median survival by 35% to 42.5 days (*P*=0.0033), indicating that AP-2α overexpression generated slower growing tumors that exhibited a longer tumor latency (12 days) than control stem cells. IHC analysis revealed that Nanog and Ki67 expression was weakly detected in tumor tissues overexpressing AP-2α (Figure 3J). These results indicated that AP-2α overexpression significantly suppresses the spheroid formation and self-renewal abilities of GSCs both *in vitro* and *in vivo*.

### AP-2α overexpression increases the chemosensitivity of glioma cells to TMZ

**Figure 4:**
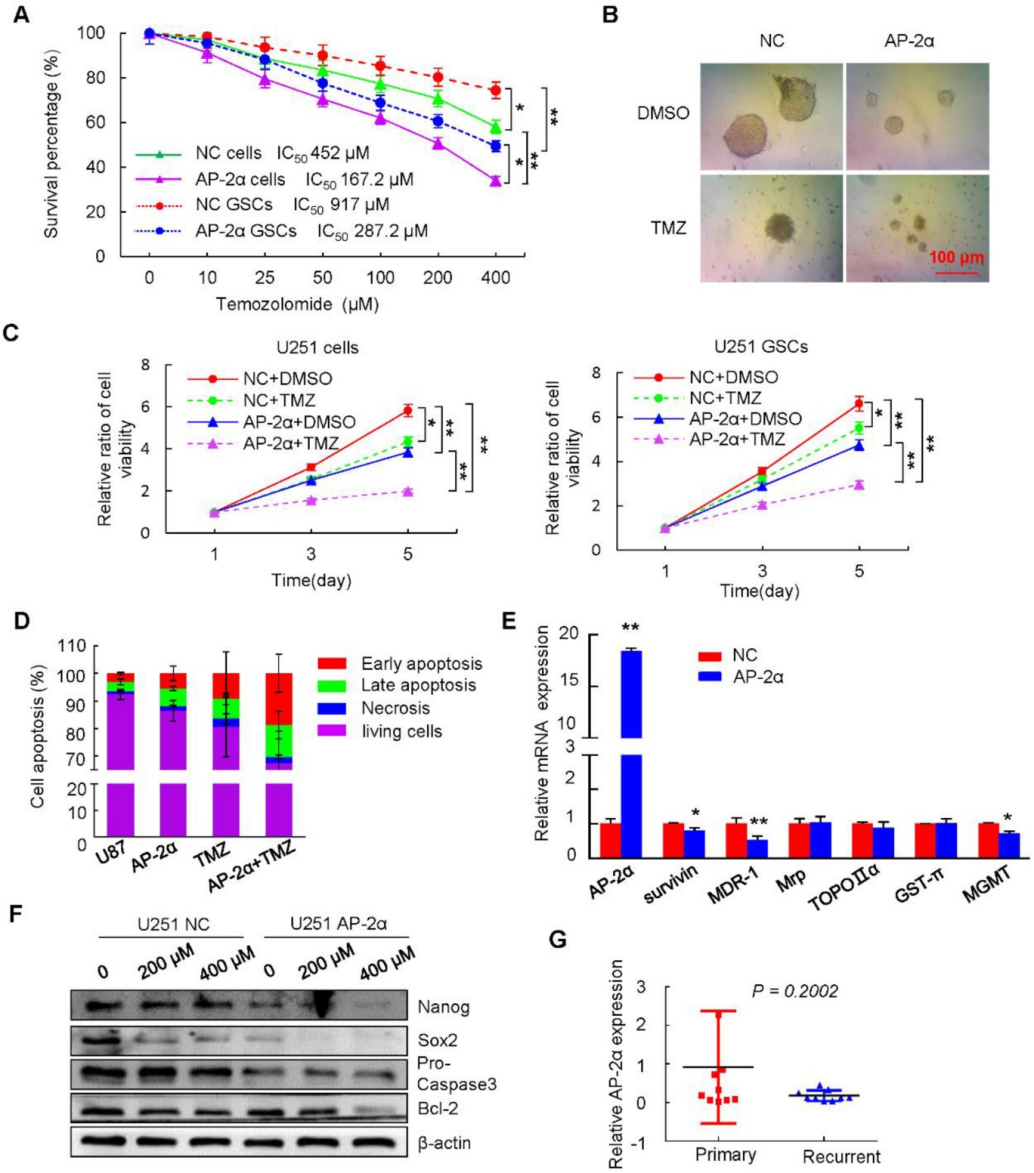
** AP-2α increases the sensitivity of glioma cells to TMZ. (A)** The survival percentage was determined by MTT assays at 490 nm. U251 cells or U251 GSCs stably expressing NC or AP-2α were exposed to TMZ over the range of 0-400 μM for 48 h followed by cell viability analysis. **(B)** Gliosphere formation assays were performed on AP-2α-overexpressing U251 cells exposed to 200 μM of TMZ or DMSO for 10 days. **(C)** MTT assays of the effects of TMZ on glioma cells and GSCs at different time points. U251 cells or U251 GSCs with NC or AP-2α expression were treated with 200 μM of TMZ or DMSO for the indicated time, cell viability was analyzed by MTT assays. **(D)** Cell apoptosis assays were performed by FACS analysis. The control and AP-2α-infected cells were treated with 400 μM of TMZ or DMSO for 48 h, cell apoptosis was then evaluated using flow cytometry. All results are expressed as the mean ± SD of three independent experiments. **(E)** qRT-PCR analysis of the effects of AP-2α on the expression of these resistance genes in U251 cells. **(F)** Western blots were performed to detect the effect of TMZ on protein levels of AP-2α downstream genes in U251 cells. **(G)** qRT-PCR analysis of AP-2α expression in 9 primary and recurrent glioma samples. *, *P* < 0.05, **, p<0.01.

AP-2α expression could sensitize cancer cells to chemotherapy and increase the sensitivity of hepatocellular cancer cells to cisplatin 22. To determine whether AP-2α sensitizes U251 cells to TMZ-induced cytotoxicity, dose-response curves for TMZ were generated alone or in combination with AP-2α overexpression, the drug concentration was determined based on when the growth of viable cells was inhibited by 50% (IC_50_). The IC_50_ value of U251 cells treated with TMZ was 452 µM, but this value was lowered to 167.2 µM in AP-2α-overexpressing U251 cells (*P*<0.01). The inhibition of sphere formation required a higher dose of TMZ, the IC_50_ value of TMZ for sphere formation inhibition was 917 µM in U251 GSCs, and only 287.2 µM in AP-2α-overexpressing U251 GSCs, indicating that AP-2α overexpression increased the sensitivity of glioma cells to TMZ (Figure 4A). Moreover, the combination of TMZ and AP-2α significantly attenuated the tumor sphere formation ability of U251 GSCs (Figure 4B). MTT assays showed that TMZ and AP-2α markedly inhibited cell proliferation, respectively. The combination of TMZ and AP-2α exhibited an even stronger inhibitory effect on the proliferation (*p*<0.01) of both glioma cells and GSCs compared to that generated by the controls (Figure 4C). Next, the apoptosis rates of AP-2α-infected and control glioma cells were examined by flow cytometry. Consistently, the apoptosis rate was enhanced by 48 h treatment with 400 μM TMZ (16.51 ± 9.85%), AP-2α overexpression (11.94 ± 3.5%), and TMZ treatment in combination with AP-2α overexpression (30.45 ± 11.79%) compared to that of control cells (6.64 ± 1.5%) (Figure 4D).

Because the development of multidrug resistance (MDR) in cancer cells is one of the major challenges to current cancer treatment efforts 39, we next examined how AP-2α influences drug resistance factors such as MDR-1 and survivin. The upregulation of AP-2α decreased the expression levels of MDR-1, survivin and MGMT as determined by quantitative RT-PCR (Figure 4E). Both TMZ and AP-2α synergistically inhibited the growth and induced the apoptosis of glioma cells, accompanied by decreased levels of Bcl-2, Pro Caspase-3 and the stem cell markers Nanog and Sox2 in a dose-dependent manner (Figure 4F). Clinically, low expression of AP-2α was detected in 9 TMZ-treated recurrent glioma patients, although there was no statistical significance compared with that in primary glioma patients (Figure 4G). These results revealed that AP-2α and TMZ synergistically decrease the proliferation and increase the chemosensitivity of glioma cells and GSCs.

### AP-2α downregulates the expression of the Nanog/Sox2/CD133 regulatory axis

**Figure 5:**
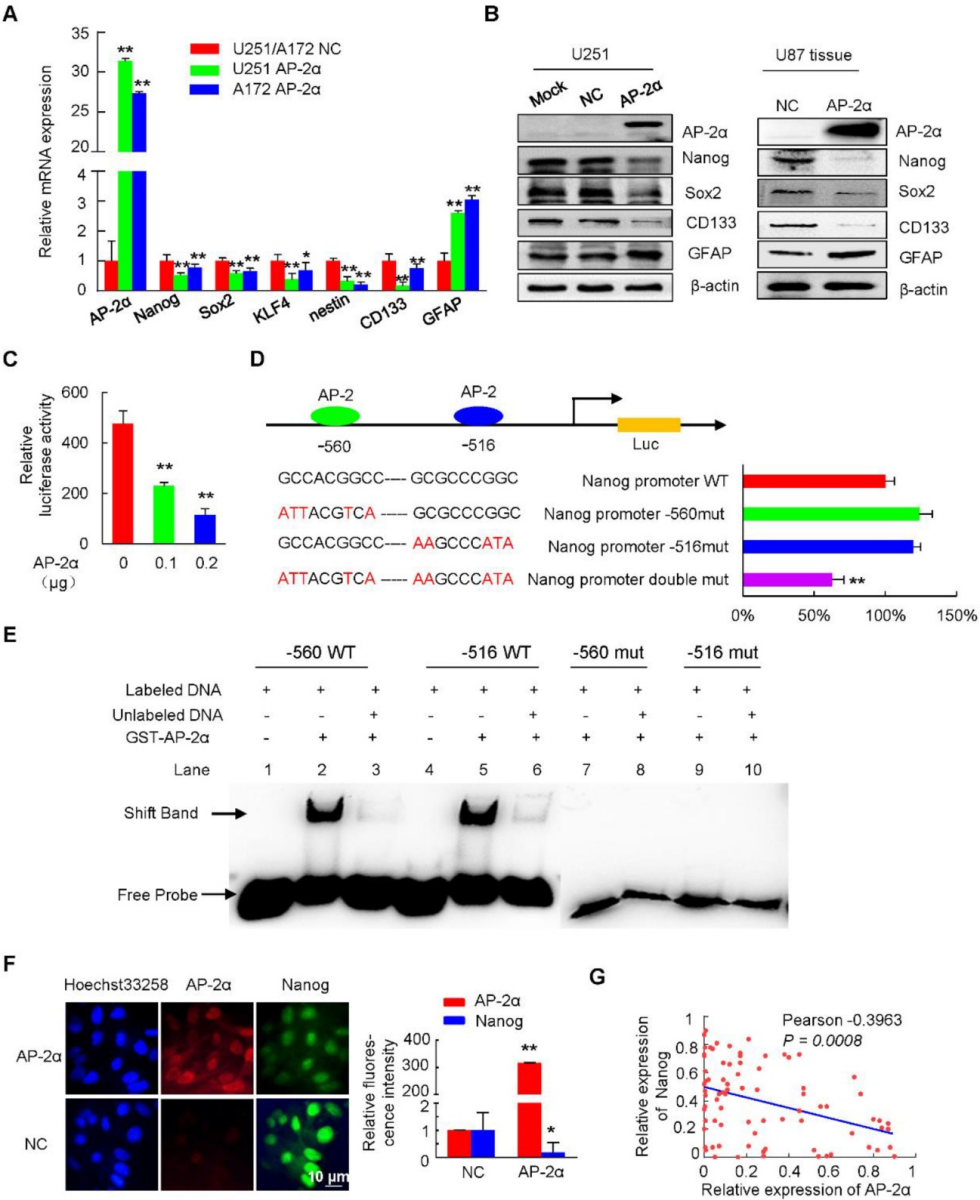
** AP-2α inhibits Nanog expression. (A)** qRT-PCR was performed to elucidate the effect of AP-2α on the expression of stem cell markers. The relative expression of each gene in the U251 AP-2α and A172 AP-2α groups was normalized to that in the U251 NC and A172 NC groups, which were set to 1 as the control. **(B)** Western blots were performed to detect the expression of stem cell markers in AP-2α-overexpressing U251 cells (left) and subcutaneous mouse tumor tissues from U87 cells (right). **(C, D)** Luciferase assays were performed to detect the effects of AP-2α on the transcriptional activities of Nanog regulatory regions, including wild-type and mutated AP-2 binding sites. **(C)** Luciferase activity was normalized to β-galactosidase activity and the results were presented as the mean ± SD of three independent transfection experiments in triplicate. **(D)** Data are presented as the fold change relative to Nanog wild-type reporter activity inhibited by AP-2α, which is considered 100%. **(E)** Electrophoretic gel mobility shift assays of AP-2α direct binding to the Nanog regulatory region *in vitro*. GST-AP-2α fusion protein was incubated with biotin-labeled AP-2 binding oligonucleotides from Nanog regulatory region. Shift band indicates protein-DNA complexes. **(F)** Immunofluorescent staining confirmed the decreased expression of Nanog in AP-2α-overexpressing U251 cells. Relative fluorescence intensity was quantified using ImageJ software. **(G)** The correlation of AP-2α and Nanog expression in glioma tissues was analyzed using GraphPad Prism. WT, wild-type. *, p<0.05, **, p<0.01, compared with controls.

As AP-2α inhibited GSCs and TMZ resistance, and negative correlation was observed between AP-2α and Nanog or Sox2, we next speculated whether AP-2α influenced the expression of stem cell-associated markers. Quantitative RT-PCR analysis showed decreased expression of Nanog, Sox2, KLF4, nestin and CD133, together with increased expression of the differentiation marker glial fibrillary acidic protein (GFAP) in AP-2α overexpressing glioma cells (Figure 5A). Moreover, Western blot analysis demonstrated that AP-2α decreased the expression of Nanog, Sox2 and CD133, and increased the levels of GFAP in both U251 cells and subcutaneous mouse tumor tissues originating from U87 cells (Figure 5B).

To further investigate the molecular mechanisms by which AP-2α regulates stem cell-related factors, we identified the potential AP-2α binding sites in the Nanog regulatory region (-741/+18) using JASPAR software and then examined the effect of AP-2α on Nanog transcription activity. The 5' regulatory regions of Nanog were amplified and inserted into pGL3-Basic luciferase reporter vector. Luciferase assays showed that AP-2α significantly suppressed Nanog transcriptional activity in a dose-dependent manner (Figure 5C). We next generated three Nanog promoter-luciferase reporters bearing mutated AP-2 binding sites. Double mutation of the AP-2 binding sites (-560 GCCACGGCC and -516 GCGCCCGGC) greatly reduced the inhibition of AP-2α-mediated Nanog reporters by 41%, while single mutation had no effect (Figure 5D). To demonstrate the direct binding of AP-2α and the putative AP-2 binding sites of the Nanog regulatory region *in vitro*, we performed EMSA experiments with purified GST-AP-2α protein and two 25-bp probes (nucleotides -527 to -503 and -568 to -544) including AP-2 binding sites. As shown in Figure 5E, strong binding was observed between the two hot probes and the AP-2α protein. The EMSA competition assays showed that the specific complexes were decreased when 50-fold of two cold 25-bp probes were used. In contrast, two mutated hot 25-bp probes could not bind to the AP-2α protein. These results indicated that the Nanog regulatory region contains AP-2 binding sites.

We next found that AP-2α and Nanog colocalized in the nuclei of U251 cells and that AP-2α decreased the fluorescence intensity of Nanog, indicating that AP-2α can downregulate Nanog protein levels (Figure 5F). Consistent with these results, the correlation between AP-2α and Nanog was further analyzed by IHC staining in glioma samples. As reported 40-42, the expression levels of Nanog (paired Student's t-test; Figure S4A and Table S2), Sox2 (Figure S4B and Table S3) and CD133 (Figure S4C and Table S4) were significantly increased in glioma tissues compared with those in normal brain tissues, and their levels were especially increased in high-grade glioma tissues. Low levels of AP-2α were primarily observed in glioma tissues with high expression of Nanog (Fisher's exact test, *P*<0.001; Table 2), Sox2 (Fisher's exact test, *P*<0.001; Table S5) and CD133 (Fisher's exact test, *P*<0.001; Table S6). Moreover, AP-2α expression was negatively correlated with the expression of Nanog (Pearson correlation coefficient, -0.3963, *P*=0.0008; Figure 5G), Sox2 and CD133 (Pearson correlation coefficient, -0.4870 and -0.4714, *P*<0.001; Figure S4D) in glioma samples. Therefore, AP-2α could function as a negative regulator of Nanog in glioma cells.

### AP-2α suppresses the IL6/STAT3 signaling pathway by inhibiting STAT3 phosphorylation

**Figure 6:**
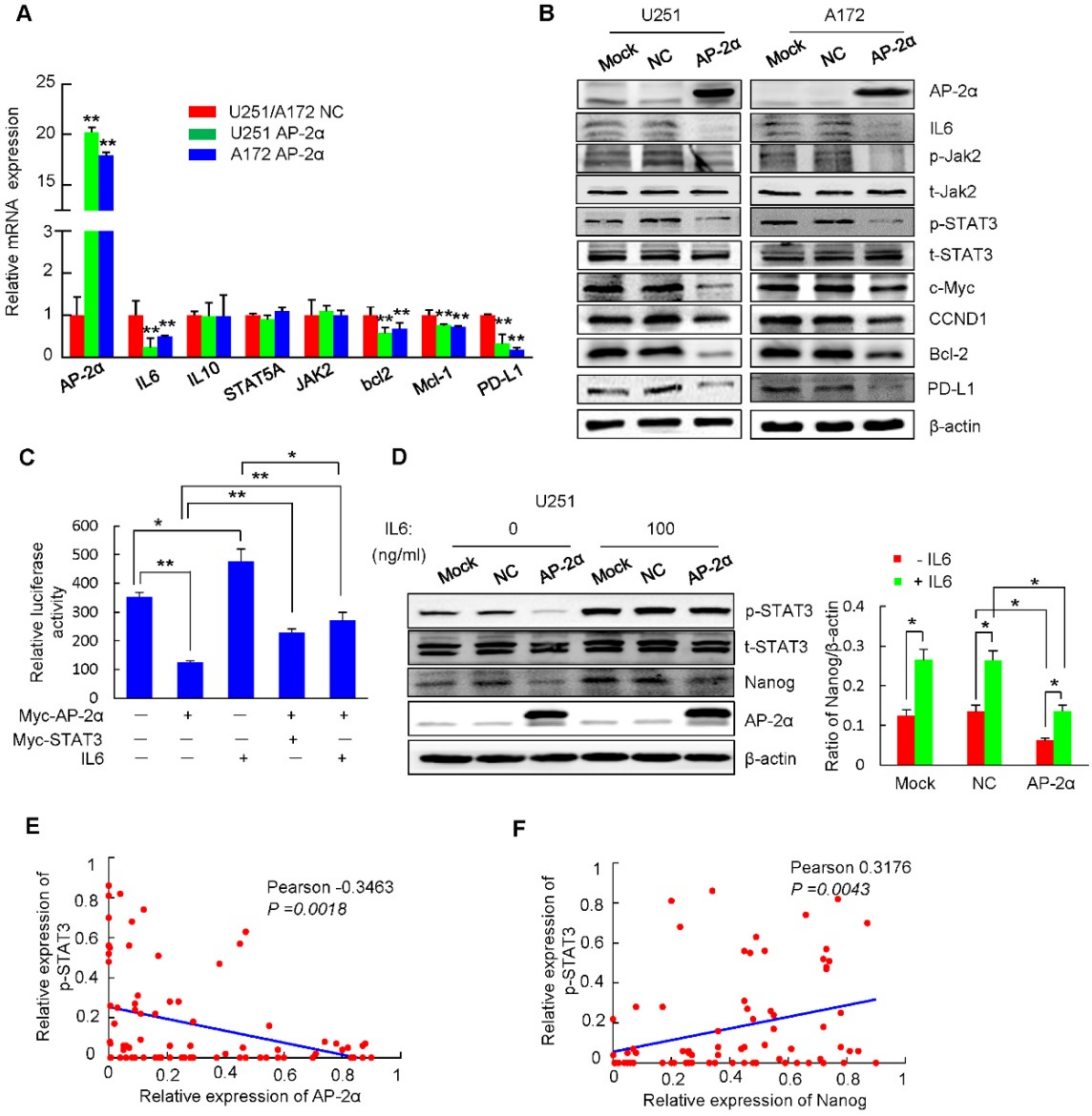
** AP-2α inhibits the IL6/STAT3 signaling pathway. (A)** qRT-PCR was performed to elucidate the effect of AP-2α on the expression of STAT3 signaling pathway-related genes. The relative expression levels of individual genes in the U251 AP-2α and A172 AP-2α groups were normalized to those in the U251 NC and A172 NC groups, which were set to 1 as the controls. **(B)** Western blots were performed to detect the expression of STAT3 signaling pathway members and STAT3 downstream genes in AP-2α-overexpressing glioma cells. **(C)** Luciferase assays were performed to detect the effects of AP-2α on the transcriptional activity of Nanog regulatory regions with or without 100 ng/mL IL6 stimulation for 8 h. Relative luciferase activity was presented as the mean ± SD of three independent transfection experiments in triplicate. **(D)** Western blots were used to detect the expression of STAT3 and Nanog in U251 cells treated or untreated with IL6 for 8 h. Relative Nanog expression was quantified by Image J software using β-actin as an internal control. **(E)** The correlation of AP-2α and p-STAT3 expression in glioma tissues was analyzed using GraphPad Prism. **(F)** The correlation of Nanog and p-STAT3 expression in glioma tissues was analyzed using GraphPad Prism. *, p<0.05, **, p<0.01, compared with controls.

STAT3 is associated with various physiological and pathological roles, and functions as a transcription activator that is phosphorylated by receptor-associated JAK and translocates to the nucleus. Clinical studies have confirmed that the continuous activation of STAT3 is closely related to the occurrence and development of glioma 43. Importantly, phosphorylated STAT3 has been shown to bind to the Nanog promoter and regulate its transcription in liver CSCs and colorectal cancer (CRC) cells 44-46. Thus, we investigated whether AP-2α regulates the STAT3 signaling pathway and mediates Nanog transcription. Quantitative RT-PCR analysis showed significantly decreased levels of the cytokine IL6 and the downstream genes Bcl-2, Mcl-1 and PD-L1 in AP-2α overexpressing U251 and A172 cells (Figure 6A). Further, Western blot also demonstrated that AP-2α overexpression significantly decreased the protein levels of IL6, activated JAK2 (p-Jak2 Y1007/1008) and STAT3 (p-STAT3 Y705), and consequently downregulated the expression of downstream proteins (c-Myc, CCND1, Bcl2 and PD-L1) (Figure 6B). We next determined whether STAT3 is involved in AP-2α-mediated Nanog expression by treating U251 cells with IL-6. Luciferase analysis demonstrated that AP-2α suppressed the transcriptional activity of the Nanog regulatory region, while IL6 stimulation enhanced Nanog reporter activity, both IL6 and STAT3 overexpression alleviated the AP-2α-mediated transcriptional inhibition of Nanog (Figure 6C). AP-2α reduced STAT3 phosphorylation in the absence of IL-6, but STAT3 phosphorylation was substantially increased in AP-2α overexpressing U251 cells treated with 100 ng/mL IL6. It is worth noting that the expression of Nanog was increased in the presence of IL6 (Figure 6D). These data suggest that the IL6/STAT3 pathway is partially involved in AP-2α-mediated Nanog expression.

As reported 47, p-STAT3 expression was significantly increased in glioblastoma tissues compared to that in normal brain tissues (paired Student's t-test; Figure S4E). The expression of p-STAT3 was negatively correlated with AP-2α expression in glioma samples (Figure 6E), in which p-STAT3 expression was observed together with low AP-2α expression (Fisher's exact test, *P*<0.001; Table S7). But the expression of p-STAT3 was positively correlated with Nanog expression in glioma samples (Figure 6F). A total of 36.0% of patients with low AP-2α expression showed high expression levels of the Nanog/p-STAT3 (*p*<0.05). Thus, AP-2α regulates Nanog expression, at least in part, dependent on the STAT3 signaling pathway.

### miR-26a binds to the 3' UTR of AP-2α, inhibits AP-2α expression, and subsequently attenuates the inhibitory effects of AP-2α on glioma

**Figure 7:**
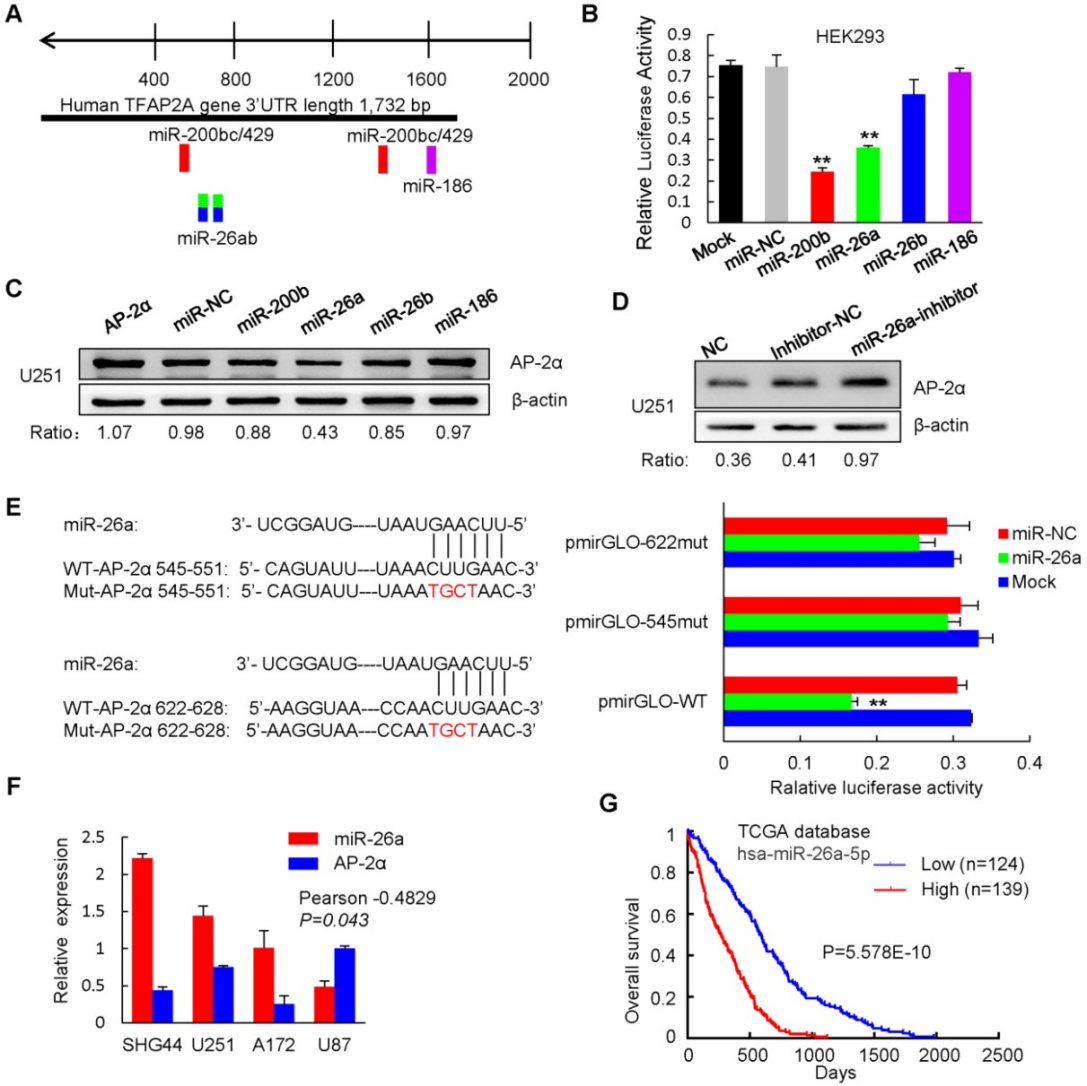
** miR-26a targets AP-2α by binding to its 3' UTR. (A)** Putative binding sites of miR-26a in the AP-2α 3'-UTR region analyzed by TargetScan. (B) miR-26a regulates the luciferase activity of the AP-2α 3'-UTR in HEK293 cells. Relative firefly luciferase reporter activity was significantly reduced when pmirGLO-AP-2α vector was cotransfected together with miR-26a mimics. Firefly luciferase activity was normalized based on Renilla luciferase activity. **(C, D)** Western blot analysis of AP-2α expression in miR-26a transfected AP-2α-overexpressing U251 cell line (C) and miR-26a inhibitor transfected U251 NC cells (D). U251 cells were collected 48 h after miRNA transfection. AP-2α expression was quantified by Image J software using β-actin as an internal control. **(E)** AP-2α 3'-UTRs with wild-type or mutated miR-26a binding sites were cotransfected with miR-26a mimics. The luciferase activity was detected. Statistical analysis was performed using SPSS software. **, p<0.01. **(F)** qRT-PCR analysis of the expression of miR-26a in glioma cell lines. The correlation between miR-26a expression and AP-2α expression in glioma cell lines was shown. **(G)** The correlation of miR-26a expression and overall survival of glioma patients was determined from The Cancer Genome Atlas (TCGA) data. n, sample number.

Previous studies have shown that the miR-200b/200c/429 family negatively regulates AP-2α expression by binding to the 3' untranslated region (UTR) of AP-2α in endometrial cancer cells 23. To identify AP-2α upstream regulators, target prediction algorithms (Targetscan) were used to predict miRNAs, and six potential miRNAs were identified (Figure 7A). We next determined whether these miRNAs regulate AP-2α by inserting the 3'-UTR sequences of AP-2α into the 3'-end of the firefly luciferase gene of the dual-luciferase miRNA target expression vector pmirGLO. Dual luciferase reporter assays showed that the miR-26a mimics inhibited the luciferase activity of AP-2α 3'-UTR vector as miR-200b induced strong activity inhibition, but neither the miR-26b mimics nor the miR-186 mimics had the inhibitory effect in HEK293 cells (Figure 7B). However, Western blot showed that only miR-26a significantly decreased AP-2α protein expression (Figure 7C), while the miR-26a inhibitor was able to rescue this suppression in U251 cells (Figure 7D). Furthermore, mutating two miR-26a binding sites in AP-2α 3' UTR led to no changes in the luciferase activity, suggesting that the predicted two miR-26a binding sites are important for miR-26a to bind AP-2α (Figure 7E). Importantly, miR-26a was highly expressed in glioblastoma cell lines and negatively correlated with AP-2α expression (Figure 7F). Moreover, miR-26a was negatively associated with the overall survival of glioma patients based on data from The Cancer Genome Atlas (TCGA) (Figure 7G). These data indicated that oncogenic miR-26a binds to the 3'-UTR of AP-2α and negatively regulates AP-2α expression in glioma.

**Figure 8:**
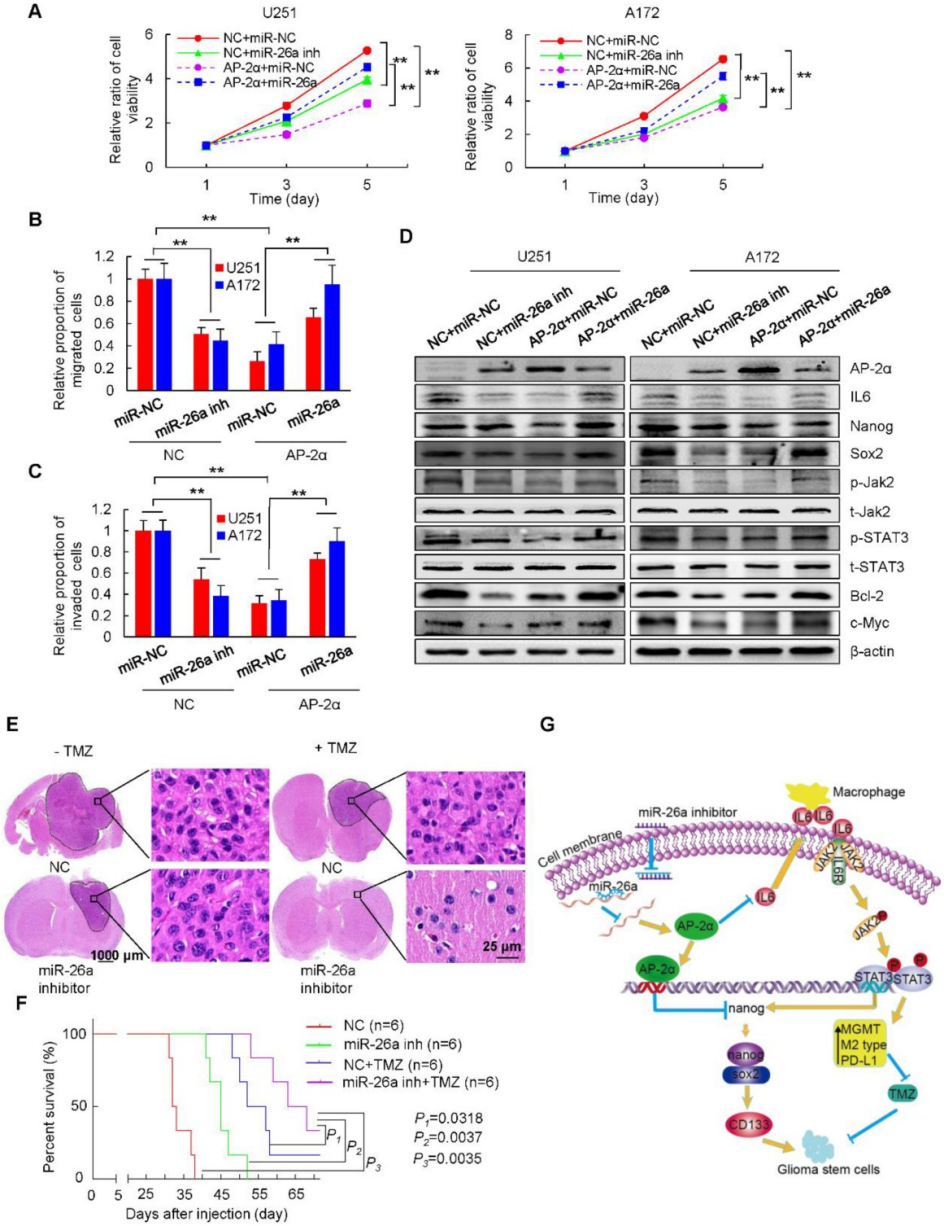
** miR-26a reverses the inhibitory effects of AP-2α on glioma cells. (A)** Glioma cells were infected with lentiviruses to overexpress AP-2α, antisense miR-26a inhibitor (miR-26a inh) or in combination with miR-26a mimics and AP-2α, and cell viability was analyzed by MTT assays. **(B, C)** Glioma cells overexpressing AP-2α or miR-26a inhibitor or with miR-26a mimics and AP-2α in combination were subjected to transwell migration and invasion assays. **(D)** Western blots were performed to detect the effect of miR-26a on the expression of AP-2α downstream genes in glioma cell lines. **(E)** U87 GSCs (3×10^5^) were injected stereotactically into the brain of BALB/c nude mice. H&E staining of brain tissues was shown in four groups, including the control, miR-26a inhibitor group, TMZ group and both miR-26a inhibitor and TMZ group. The volume of the intracranial tumors was measured as mean ± SD. **(F)** Kaplan-Meier survival curves of nude mice bearing intracranial tumors of U87 GSCs in four groups. All data are presented as the mean ± SD of three independent experiments. **, p<0.01, compared with the control group. **(G)** Schematic presentation of the mechanism underlying AP-2α-suppressed glioma stemness and TMZ resistance.

miR-26a functions as an oncogene in a variety of cancers, and its overexpression is associated with the poor prognosis of cancer patients 48. We further dissected the role of miR-26a in AP-2α-mediated glioma development. The miR-26a inhibitor was completely complementary to miR-26a and suppressed miR-26a activity, resulting in increased levels of the miR-26a target AP-2α and thus inhibited glioma cell growth just as AP-2α overexpression did. However, miR-26a mimics significantly alleviated the inhibitory proliferation of AP-2α-overexpressing glioma cells (Figure 8A). The migration and invasion capacities of glioma cells were notably blocked by AP-2α or the miR-26a inhibitor, but enhanced in the presence of miR-26a mimics (Figure 8B-C). Moreover, miR-26a alleviated the inhibitory effect of AP-2α and the miR-26a inhibitor on glioma cells by upregulating the expression of Nanog/Sox2 and the IL6/Jak2/STAT3 signaling pathway (Figure 8D). Collectively, these results suggested that AP-2α is a direct target of miR-26a, and the miR-26a inhibitor can upregulate AP-2α expression and function as a tumor suppressor in glioma cells.

Finally, we utilized intracranial mouse models to analyze the synergistic effect of the miR-26a inhibitor and TMZ on glioma development. We found that the miR-26a inhibitor (26.41 ± 15.04 mm^3^) and TMZ (21.81 ± 8.51 mm^3^) alone reduced the intracranial tumor volume by about 62.6% and by approximately 69.1%, respectively, compared with that of tumors generated from NC stem cells (70.58 ± 30.31 mm^3^), while the combination of both (5.69 ± 4.64 mm^3^) synergistically decreased the tumor volume even more significantly by 91.9% (*p*<0.05) (Figure 8E). The survival time of intracranial xenograft mice was prolonged by the miR-26a inhibitor, TMZ and the TMZ/miR-26a inhibitor combination as determined by Kaplan-Meier analysis (Figure 8F). The median survival time of mice in the control group was 32.5 days. In contrast, the median mouse survival time was increased by 38.5% to 45 days for miR-26a inhibitor treatment, by 60% to 52 days for TMZ treatment, and by 102 % to 65.5 days for TMZ/miR-26a inhibitor combination (*P*=0.0035). These data indicated that the miR-26a inhibitor and TMZ synergistically decreased tumor growth and prolonged longer tumor latency (22 days) compared with those of tumors generated from control cells.

## Discussion

AP-2α, a central member of the AP-2 family with tissue specificity and DNA binding specificity 49, 50, plays an important role in cell differentiation and tumorigenesis 51. In the present study, we observed low AP-2α expression in 58.5% of glioma samples, and AP-2α expression was negatively associated with patient age (*p*<0.05), tumor diagnosis and tumor grade (*p*<0.0001). The negative correlation between AP-2α and Nanog expression, Sox2 expression, CD133 expression, and p-STAT3 expression was further confirmed in glioma tissues and cell lines. Moreover, 72.0% of patients with decreased AP-2α expression showed enhanced Nanog levels, while 78.0% and 58.0% of patients with decreased AP-2α showed increased Sox2 and CD133 expression, respectively (*p*<0.001). A total of 44.0% of patients with decreased AP-2α expression showed consistently enhanced expression levels of the Nanog/Sox3/CD133 axis (Table 3, *p*<0.05). These results indicated that the combination of AP-2α with these downstream genes might provide novel molecular approaches for the clinical diagnosis of glioma and individualized therapy for glioma patients.

Cancer stem cells (CSCs) are defined as cells with tumor-initiating capacity, self-renewal ability, and differentiated progeny 52, 53. Malignant glioma contains a population of glioblastoma stem cells (GSCs), which play important roles in self-renewal, gliosphere formation, proliferation, invasion, angiogenesis and immune response 54, 55. Some molecule markers are associated with the maintenance of GSCs, such as the cell surface markers CD133, CD15, the cytoskeletal protein Nestin 38, 56, 57 and transcriptional factors in embryonic stem cells (ESCs), such as Nanog, Oct-4, Sox2, and Klf4, which control pluripotency and differentiation 58-61. Our results presented herein show that AP-2α suppresses the proliferation of glioma cells and the sphere formation and self-renewal abilities of GSCs. Further clinical data confirmed that Nanog expression was increased and negatively correlated with AP-2α in glioma tissues. Mechanically, we found that AP-2α binds to the 5′ regulatory region of the Nanog gene, decreases its transcription and protein levels, and further downregulates the expression of the interacting partner Sox2 62. Decreased Sox2 markedly decreased CD133 expression in glioma cells, as previously reported 37. Subsequently, AP-2α decreased the proportion of CD133-positive glioma cells and inhibited tumor self-renewal in CD133^+^ glioma cells. Therefore, as a potential tumor suppressor, AP-2α inhibits the tumor sphere formation, self-renewal and intracranial tumor formation capacities of GSCs by regulating the Nanog/Sox2/CD133 signaling axis, suggesting that AP-2α is responsible for the maintenance of stem cell-like tumor growth, and could serve as a prime therapeutic target for the treatment of glioma.

GSCs are responsible for drug resistance and tumor recurrence in patients with glioma. The alkylating agent TMZ methylates the nucleotide units of DNA and causes the formation of an abnormal O6-MG/T match, which activates the DNA mismatch repair (MMR) system 63 and results in unrepairable DNA damage followed by glioma cell cycle arrest and apoptosis 64. However, a considerable number of GBM patients are refractory to TMZ, and more effective therapeutic options are urgently needed. AP-2α inhibits cell growth by inducing cell cycle arrest and apoptosis 8, 9, 65-67 and increases the chemosensitivity of hepatocellular carcinoma cells to cisplatin 22. We found that overexpression of AP-2α significantly increased glioma cell apoptosis and synergistically enhanced the cytotoxicity of TMZ, which was in concomitant with decreased expression of Survivin, a critical drug resistance regulator, and the downstream gene MDR-1. AP-2α also inhibits the DNA repair protein MGMT, which renders cells resistant to the cytotoxic actions of methylating and chloroethylating agents, such as TMZ 68. As AP-2α was shown to regulate GSCs and TMZ resistance in recurrent gliomas, we also demonstrated that AP-2α was expressed at low levels in glioma tissues from nine TMZ-resistant recurrence patients, suggesting that TMZ resistance downregulated AP-2α expression. Conversely, AP-2α overexpression enhanced TMZ sensitivity. In summary, a novel therapeutic strategy combining TMZ with AP-2α could be an efficient way to induce glioma apoptosis and suppress tumor recurrence.

GBMs reportedly display a significantly higher level of IL-6 expression and STAT3 activity than normal brain tissue 69, 70. STAT3 activation is significantly associated with stem-like GBM cells 71. STAT3 activation was shown to be necessary for increased levels of MGMT in GBM, while STAT3 inhibition downregulated MGMT expression to overcome TMZ resistance in GBM 72. Our current results showed that AP-2α could downregulate IL6 expression and block the Jak2/STAT3 signaling pathway in glioma. Because phosphorylated STAT3 could bind to the murine Nanog promoter and activate its transcription in ESCs 45, we also demonstrated that the expression of Nanog was slightly increased in the presence of IL6 in AP-2α overexpressing glioma cells, suggesting that STAT3 is partially involved in AP-2α-mediated Nanog expression and that certain overlaps and crosslinks exist between glioma stemness and drug resistance. In addition, glioma-derived IL-6, together with other tumor-secreted factors, such as TGFβ, polarize glioma-infiltrating microglia toward the immunosuppressive M2 phenotype through STAT3 activation, which increases the expression of the immunosuppressive factors TGF-β2, IL-1 and the costimulatory molecules CD80/86 73-76 and confers high PD-L1 expression to promote tumor immune evasion 77. AP-2α was found to suppress classically activated (M1) macrophages producing the cytokine IL-6 and Jak2/STAT3 activation and subsequently decrease PD-L1 expression in glioma cells. The expression of PD-L1 is correlated with the tumor grade of glioma patients, and high levels of PD-L1 promote the growth of most malignant gliomas and facilitate immune evasion 78. Thus, AP-2α functions as a bridge among stemness, drug resistance and immune evasion. AP-2α inhibits the stemness of GSCs, synergistically decreases TMZ resistance, improves anticancer chemotherapies and leads to the avoidance of immune evasion (Figure 8G). Elucidating the interplay between AP-2α and inflammatory cytokines and immune cells in glioma is important, as it will facilitate our understanding of AP-2α function and reveal the clinical importance of AP-2α in combination strategies for treating gliomas.

Oncogenic miR-26a was shown to be highly expressed in glioma cell lines 79, while miR-200b was significantly downregulated in glioma tissues 80. Although miR-200b binds to the 3′ UTR of AP-2α, miR-200b could not specifically regulate AP-2α expression in glioma. miR-26a binds to the 3′ UTR of AP-2α, inhibits its expression, and decreases its tumor suppressive effects. miRNA mimic delivery is a feasible strategy based on substantial amounts of preclinical data from animal models, miR-34 was the first miRNA to advance into the clinic for the treatment of patients with primary or metastatic liver cancer 81. The miR-10b antagonist was preclinically developed to treat glioblastoma 82. The miR-26a inhibitor complemented miR-26a to increase the expression of AP-2α and decrease downstream Nanog and STAT3 expression. The miR-26a inhibitor generated tumors that grew slower and had a longer tumor latency (10 days) than those generated by control. Moreover, the miR-26a inhibitor and TMZ combinedly inhibited glioma progression, and prolonged the median survival time from 32.5 days to 65.5 days.

## Conclusions

AP-2α functions as a novel tumor suppressor to inhibit the stemness of glioma cancer cells by inhibiting the Nanog/Sox2/CD133 regulatory axis and decreasing the resistance of glioma cells to TMZ by blocking the IL6/Jak2/STAT3 signaling pathway. Additionally, AP-2α expression is downregulated by oncogenic miR-26a, and inhibiting miR-26a represents as a novel factor for synergistic therapy with TMZ. Therefore, understanding the underlying mechanisms of AP-2α in glioma has new implications in future therapies to inhibit glioma progression and recurrence.

## Supplementary Material

Supplementary figures and tables.

## Figures and Tables

**Table 1:** AP-2α expression and clinical characteristics

Clinical features	Number	Overexpression	Low expression	P value
Total number	147			
Gender				0.697
Female	43	19	24	
Male	87	36	51	
Age (median, 39 years)				0.036
<	62	13	49	
≥	68	42	26	
Histological diagnosis				<0.0001
Astrocytoma	49	38	11	
Glioblastoma	81	17	64	
Histological grade				<0.0001
Grade I/II	35	32	3	
Grade III/IV	95	23	72	
Normal tissue	17			

**Table 2:** Correlation between AP-2α expression and Nanog expression in glioma samples by IHC analysis.

AP-2α expression	Cases	Nanog expression		P value*
		Low, No (%)	High, No (%)	
Low (≤30%)	50	14 (28.0%)	36 (72.0%)	
High (>30%)	36	26 (72.2%)	10 (27.8%)	<0.001
Total	86	40 (46.5%)	46 (53.5%)	

All 86 samples were divided according to the proportion score to define AP-2α or Nanog expression with low or high staining. *Fisher's exact test.

**Table 3:** Correlation between AP-2α expression and Nanog/Sox2/CD133 expression in glioma samples by IHC analysis.

AP-2α expression	Cases	Nanog /Sox2/CD133 expression		P value*
		Low, No(%)	High, No(%)	
Low(≤30%)	50	6(12.0%)	22(44.0%)	0.012
High(>30%)	36	21(58.3%)	3(8.33%)	
Total	86	27(31.4%)	25(29.1%)	

*ANOVA.

## Abbreviations

ATCC: American Type Culture Collection
Bcl2: B-cell lymphoma 2
bFGF: basic fibroblast growth factor
CCND1: cyclin D1
DMEM: Dulbecco's Modified Eagle Medium
DMSO: dimethylsulfoxide
EGF: Epidermal growth factor
EMSA: electrophoretic mobility shift assay
FACS: fluorescence-activated cell sorting
FBS: fetal bovine serum
GAPDH: Glyceraldehyde 3-phosphate dehydrogenase
GBM: glioblastoma multiforme
GFAP: glial fibrillary acidic protein
GSCs: glioblastoma stem cells
GST-π: glutathione S-transferase π
H&E: hematoxylin and eosin
IB: immunoblotting
IC50: the half maximal inhibitory concentration
IF: immunofluorescence
IHC: immunohistochemistry
IL6: interleukin 6
JAK2: janus kinase 2
KLF4: Kruppel-like factor 4
LEF: lymphoid enhancer factor
LDA: limiting dilution assay
Mcl-1: myeloid cell leukemia sequence 1
MDR1: multidrug resistance protein 1
MGMT: O6-methylguanine methyltransferase
MOI: multiplicity of infection
Mrp: multidrug resistance protein
MTT: 3-(4,5-dimethyNCthiazol-2-yl)-25-diphenyltetrazolium bromide
NC: negative control
PBS: phosphate-buffered saline
PD-L1: Programmed death-ligand 1
PET: polyethylene terephthalate
PFA: paraformaldehyde
PI: propidium iodide
RIPA: radioimmunoprecipitation assay
qRT-PCR: quantitive real-time polymerase chain reaction
SD: standard deviation
STAT3: signal transducer and activator of transcription 3
TCGA: Cancer Genome Atlas
TMZ: temozolomide
TOPOIIα: DNA topoisomerase II alpha
UTR: untranslated region
VEGF: vascular endothelial growth factor
