# Supplementary material for: The miR-26a/AP-2α/Nanog signaling axis mediates stem cell self-renewal and temozolomide resistance in glioma
Source: Theranostics. 2019 Jul 28;9(19):5497–516. doi: 10.7150/thno.33800 (PMC6735392; doi:10.7150/thno.33800)
Supplement: Supplementary file 1 — Supplementary figures and tables. [file thnov09p5497s1.pdf]

Supplementary Materials

Table S1 Primer pairs in this study

Name	Sequence (5'- to -3')	Purpose
AP-2α-F	AGGTCAATCTCCCTACACGAG	
AP-2α-R	GGAGTAAGGATCTTGCGACTGG	RT-PCR
AP-2α3'UTR545-551 mut-F	TGAATAAATGCTAACACAAATAC	
AP-2α3'UTR545-551 mut-R	GTATTTGTGTTAGCATTTATTCA	PCR
AP-2α3'UTR622-628 mut-F	AGCGACCAATGCTAACTTTCTC	
AP-2α3'UTR622-628 mut-R	GAGAAAGTTAGCATTGGTCGCT	PCR
BCL2-F	GGTGGGGTCATGTGTGTGG	
BCL2-R	CGGTTCAGGTACTCAGTCATCC	RT-PCR
CD133-F	AGTCGGAAACTGGCAGATAGC	
CD133-R	GGTAGTGTTGTACTGGGCCAAT	RT-PCR
GFAP-F	AGGTCCATGTGGAGCTTGAC	
GFAP-R	GCCATTGCCTCATACTGCGT	RT-PCR
GST-π-F	CCCTACACCGTGGTCTATTTCC	
GST-π-R	CAGGAGGCTTTGAGTGAGC	RT-PCR
IL6-F	CCTGAACCTTCCAAAGATGGC	
IL6-R	TTCACCAGGCAAGTCTCCTC	RT-PCR
IL10-F	TCAAGGCGCATGTGAACTCC	
IL10-R	GATGTCAAACTCACTCATGGCT	RT-PCR
JAK2-F	AGCCTATCGGCATGGAATATCT	
JAK2-R	TAACACTGCCATCCCAAGACA	RT-PCR
Klf4-F	CGGACATCAACGACGTGAG	
Klf4-R	GACGCCTTCAGCACGAACT	RT-PCR
MCL1-F	TGCTTCGGAAACTGGACATCA	
MCL1-R	TAGCCACAAAGGCACCAAAAG	RT-PCR
MDR-1-F	GGGATGGTCAGTGTTGATGGA	
MDR-1-R	GCTATCGTGGTGGCAAACAATA	RT-PCR
MGMT-F	ACCGTTTGCGACTTGGTACTT	
MGMT-R	GGAGCTTTATTTCGTGCAGACC	RT-PCR
miR-26a	GTCGTATCCAGTGCAGGGTCCGAGGTATTCGCACTGGAT ACGACAGCCTA	RT
miR-26a-F	GCGCTTCAAGTAATCCAG	
miR-26a-R	TATCCAGTGCAGGGTCC	RT-PCR
Mrp-F	CTCTATCTCTCCCGACATGACC	
Mrp-R	AGCAGACGATCCACAGCAAAA	RT-PCR
Nanog-F	CCCCAGCCTTTACTCTTCCTA	
Nanog-R	CCAGGTTGAATTGTTCCAGGTC	RT-PCR
Nanog(-741/+18)-F	CGG GGTACC ATCGGCTCACCACAACCTCT	
Nanog(-741/+18)-R	CCC AAGCTT AGCTGGATCCACACTCATGT	promoter
Nanog(-741/+18)-516 mut F	TGGGCCACCAAGCCCATACTTTTTCTTA	
Nanog(-741/+18)-516mut R	TAAGAAAAAGTATGGGCTTGGTGGCCCA	PCR
Nanog(-741/+18)-560 mut F	TGATCCGCCTATTACGTCATCCCAATTTA	

Nanog(-741/+18)-560mut R	TAAATTGGGATGACGTAATAGGCGGATCA	PCR
Nanog(-568/-544)-F	GATCCGCCTGCCACGGCCTCCCAAT	
Nanog(-568/-544)-R	ATTGGGAGGCCGTGGCAGGCGGATC	EMSA
Nanog (-527/-503)-F	GGTGGGCCACCGCGCCCGGCCTTTT	
Nanog(-527/-503)-R	AAAAGGCCGGGCGCGGTGGCCCACC	EMSA
Nanog(-568/-544)mut-F	GATCCGCCTATTACGTCATCCCAAT	
Nanog(-568/-544)mut-R	ATTGGGATGACGTAATAGGCGGATC	EMSA
Nanog(-527/-503)mut-F	GGTGGGCCACCAAGCCCATACTTTT	
Nanog(-527/-503)mut-R	AAAAGTATGGGCTTGGTGGCCCACC	EMSA
Nestin-F	CTGCTACCCTTGAGACACCTG	
Nestin-R	GGGCTCTGATCTCTGCATCTAC	RT-PCR
PD-L1-F	TGGCATTTGCTGAACGCATTT	
PD-L1-F	TGCAGCCAGGTCTAATTGTTTT	RT-PCR
pmir GLO-F	GTGGTGTTGTGTTCGTGGAC	
pmir GLO-R	TCACTGCATTCTAGTTGTGGTTT	PCR
Sox2-F	TACAGCATGTCCTACTCGCAG	
Sox2-R	GAGGAAGAGGTAACCACAGGG	RT-PCR
STAT5A-F	GCAGAGTCCGTGACAGAGG	
STAT5A-R	CCACAGGTAGGGACAGAGTC	RT-PCR
Survivin-F	AGGACCACCGCATCTCTACAT	
Survivin-R	AAGTCTGGCTCGTTCTCAGTG	RT-PCR
TopoIIa-F	TGGCTGTGGTATTGTAGAAAGC	
TopoIIa-R	TTGGCATCATCGAGTTTGGGA	RT-PCR
miR-26a	GGCUGUGGCUGGAUUCAAGUAAUCCAGGAUAGGCUGUU UCCAUCUGUGAGGCCUAUUCUUGAUUACUUGUUUCUGG AGGCAGCU	clone
miR-26a inhibitor	ccggAGCCTATCCTGGATTACTTGAATTTTTg	clone

Table S2 Nanog expression and clinical characteristics

**Clinical features**	**Number**	**Overexpression**	**Low expression**	**P value**
Total number	86			
Gender				0.7076
Female	26	17	9	
Male	49	30	19	
Age（median，39 years）				0.4344
<	32	21	11	
≥	43	26	17	
Histological diagnosis				**0.0002**
Astrocytoma	28	11	17	
Glioblastoma	47	36	11	
Histological grade				**＜0.0001**
Grade I/II	23	7	16	
Grade III/IV	52	40	12	
Normal tissue	11			

Table S3 Sox2 expression and clinical characteristics

**Clinical features**	**Number**	**Overexpression**	**Low expression**	**P value**
Total number	86			
Gender				0.7956
Female	26	17	9	
Male	49	32	17	
Age（median，39 years）				0.1655
<	32	17	15	
≥	43	32	11	
Histological diagnosis				**＜0.0001**
Astrocytoma	28	10	18	
Glioblastoma	47	39	8	
Histological grade				**＜0.0001**
Grade I/II	23	7	16	
Grade III/IV	52	42	10	
Normal tissue	11			

Table S4 CD133 expression and clinical characteristics

**Clinical features**	**Number**	**Overexpression**	**Low expression**	**P value**
Total number	86			
Gender				0.8725
Female	26	11	15	
Male	49	26	23	
Age（median，39 years）				0.5924
<	32	16	16	
≥	43	21	22	
Histological diagnosis				**0.0124**
Astrocytoma	28	10	18	
Glioblastoma	47	27	20	
Histological grade				**0.0011**
Grade I/II	23	6	17	
Grade III/IV	52	31	21	
Normal tissue	11			

Table S5 Correlation between AP-2α expression and Sox2 expression in glioma samples by IHC analysis.

**AP-2α expression**	**Cases**	**Sox2 expression**		**P value***
		**Low，No（%）**	**High，No（%）**	
Low（≤30%）	50	11（22.0%）	39（78.0%）	
High（＞30%）	36	25（69.4%）	11（30.5%）	**＜0.001**
Total	86	36（41.9%）	50（58.1%）	

All 86 samples were divided according to the proportion score to define AP-2α or Sox2 expression with low or high staining.

*Fisher's exact test.

Table S6 Correlation between AP-2α expression and CD133 expression in glioma samples by IHC analysis.

**AP-2α expression**	**Cases**	**CD133 expression**		**P value***
		**Low，No（%）**	**High，No（%）**	
Low（≤30%）	50	21（42.0%）	29（58.0%）	
High（＞30%）	36	28（77.8%）	8（22.2%）	**＜0.001**
Total	86	49（56.9%）	37（43.0%）	

All 86 samples were divided according to the proportion score to define AP-2α or CD133 expression with low or high staining.

*Fisher's exact test.

Table S7 Correlation between AP-2α expression and p-STAT3 expression in glioma samples by IHC analysis.

**AP-2α expression**	**Cases**	**p-STAT3 expression**		**P value***
		**-，No（%）**	**+，No（%）**	
Low（≤30%）	50	16（32.0%）	34（68.0%）	
High（＞30%）	36	20（55.5%）	16（44.4%）	**＜0.001**
Total	86	49（56.9%）	37（43.0%）	

All 86 samples were divided according to the proportion score to define AP-2α with low or high staining and p-STAT3 with or without staining.

*Fisher's exact test.

Figure S1

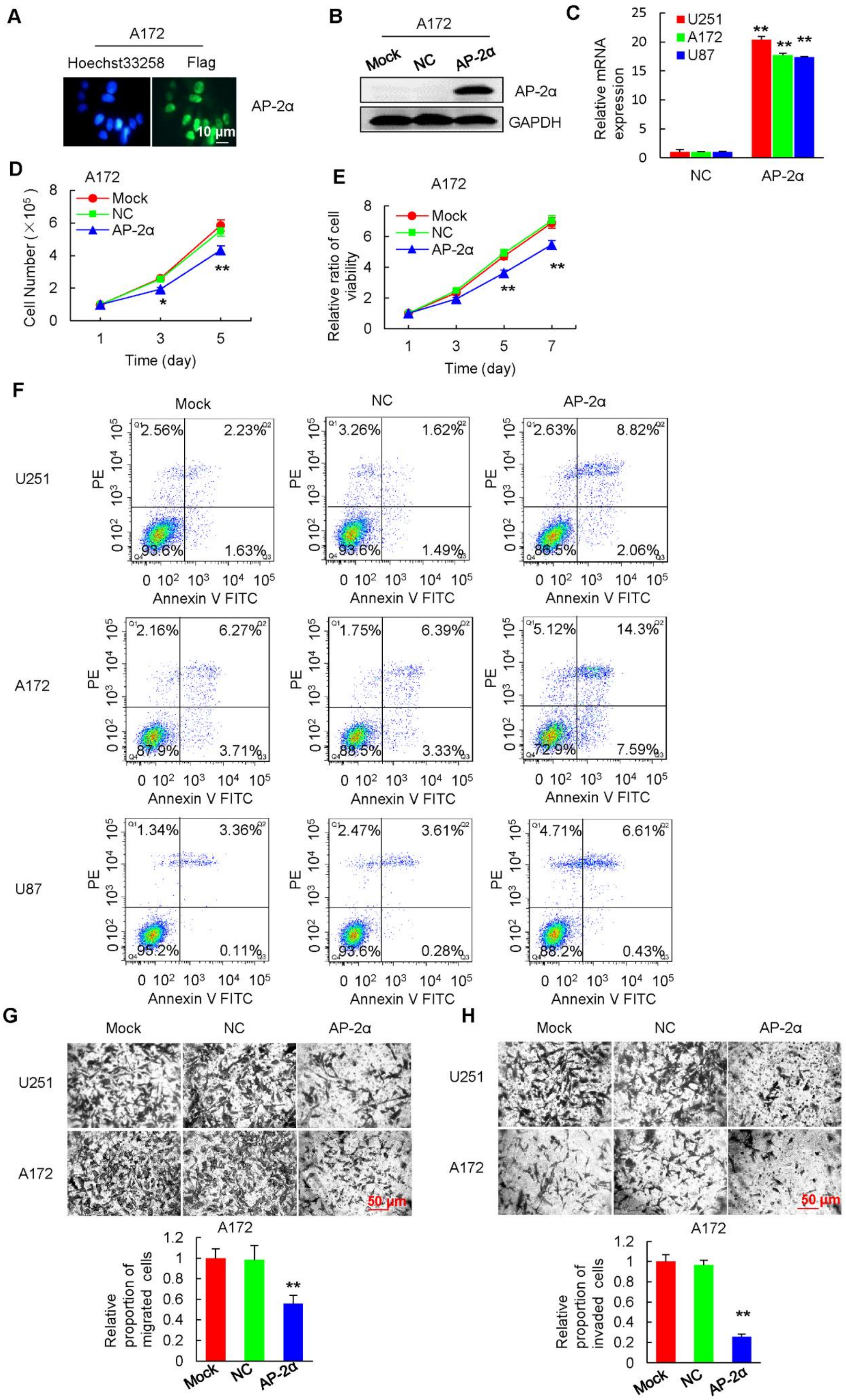
A
A172
Hoechst33258
Flag
AP-2α
10 μm
B
A172
Mock
NC
AP-2α
AP-2α
GAPDH
C
U251
A172
U87
Relative mRNA expression
NC
AP-2α
D
A172
Mock
NC
AP-2α
Cell Number (×10⁵)
Time (day)
E
A172
Relative ratio of cell viability
Time (day)
F
Mock
NC
AP-2α
U251
A172
U87
PE
Annexin V FITC
2.56%
2.23%
93.6%
1.63%
3.26%
1.62%
93.6%
1.49%
2.63%
8.82%
86.5%
2.06%
2.16%
6.27%
87.9%
3.71%
1.75%
6.39%
88.5%
3.33%
5.12%
14.3%
72.9%
7.59%
1.34%
3.36%
95.2%
0.11%
2.47%
3.61%
93.6%
0.28%
4.71%
6.61%
88.2%
0.43%
G
H
50 μm
Relative proportion of migrated cells
Relative proportion of invaded cells

Figure S1. Effects of AP-2α overexpression on glioma cell growth *in vitro*. (A) Immunofluorescence staining of Flag-AP-2α overexpression in lentiviral-infected A172 cells. (B) Western blots of Flag-AP-2α expression in A172 cells. GAPDH was served as a loading control. (C) qRT-PCR analysis of AP-2α expression in lentiviral-infected glioma cells. (D) Cell survival assays of lentiviral-infected A172 cells. (E) MTT assays of lentiviral-infected and parental A172 cells. Stable cells (5,000) were plated in octuplicate in 48-well plates and grown in DMEM with 10% FBS. The absorbance was analyzed for 1, 3, 5 and 7 days. (F) Cell apoptosis assays were evaluated using flow cytometry in the control and AP-2α-infected glioma cells. (G) Effect of AP-2α overexpression on glioma cell migration by transwell assays. Examples of migrated cells and the relative invasion proportion of A172 cells are shown. (H) Effect of AP-2α overexpression on glioma cell invasion through Matrigel. Examples of invaded cells and the relative invasion proportion of A172 cells are shown. *, $p<0.05$, **, $p<0.01$.

Figure S2

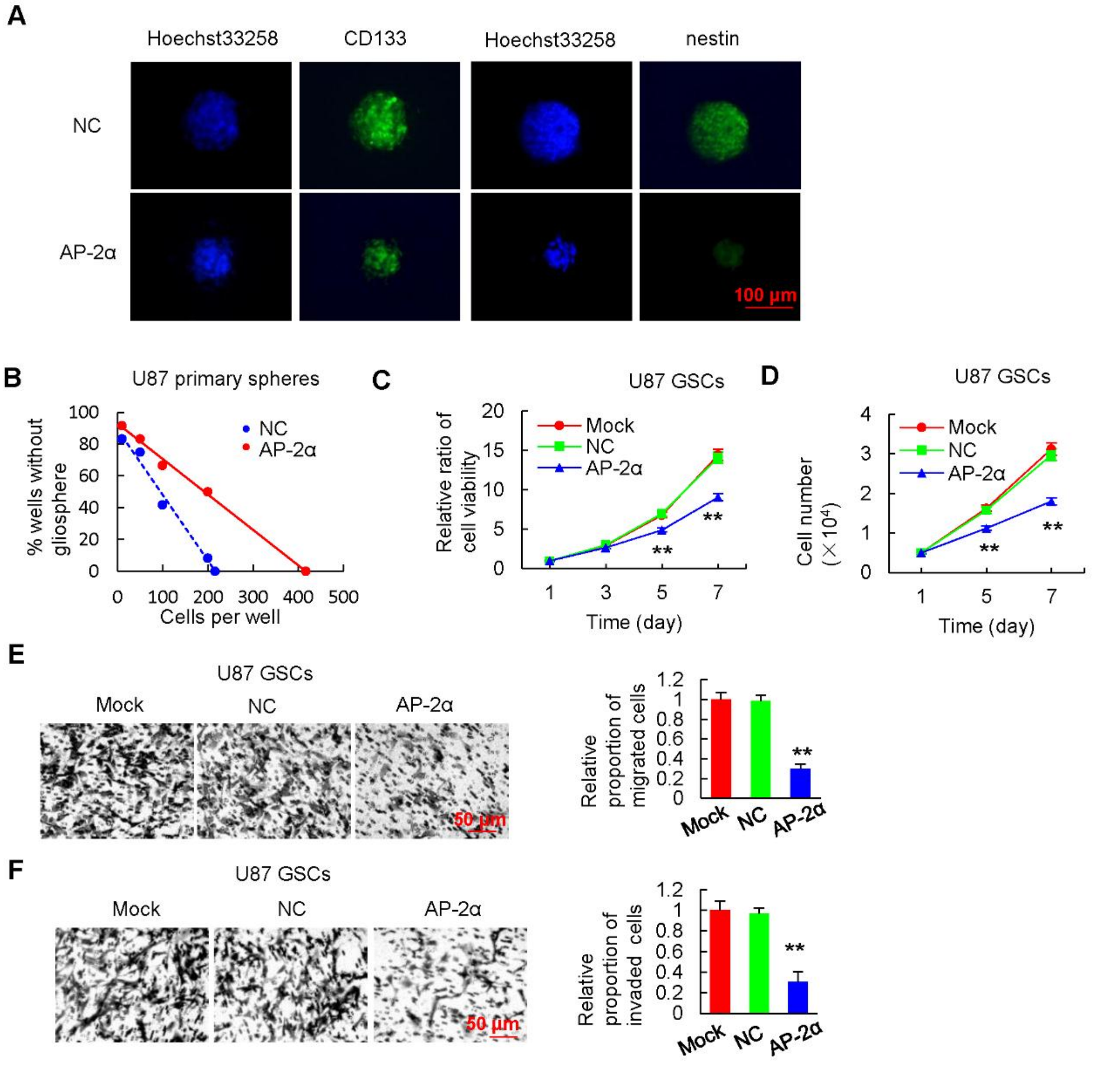

Figure S2. Effects of AP-2α overexpression on glioma stem cells *in vitro*. (A) Immunofluorescence staining showed the expression of stem marker genes CD133 and nestin in U251 GSCs. (B) A limiting dilution assay was performed to measure tumor sphere formation. Cells were plated at 200, 100, 50, 20, 10, or 5 cells/well and cultured in stem cell-conditioned medium (n = 48 wells/condition, $p = 0.049$). (C) MTT assays of lentiviral-infected and parental U87 GSCs. Stable cells (5,000) were plated in octuplicate in 48-well plates and grown in stem cell-conditioned medium. The absorbance was analyzed for 1, 3, 5 and 7 days. (D) Cell survival assays of lentiviral-infected and parental U87 GSCs. Stable cells (100,000) were plated into 6-well plates in triplicate, grown in stem cell-conditioned medium for 7 days, cell numbers were

counted with a hemocytometer. (E) Effect of AP-2α overexpression on U87 GSC migration by transwell assays. Examples of cells migrated through the PET-membrane and relative migration proportion of cells are shown. (F) Effect of AP-2α overexpression on U87 GSC invasion through Matrigel. Examples of cells migrated through Matrigel-coated Transwell and relative invasion proportion of cells are shown. **, $p<0.01$.

Figure S3

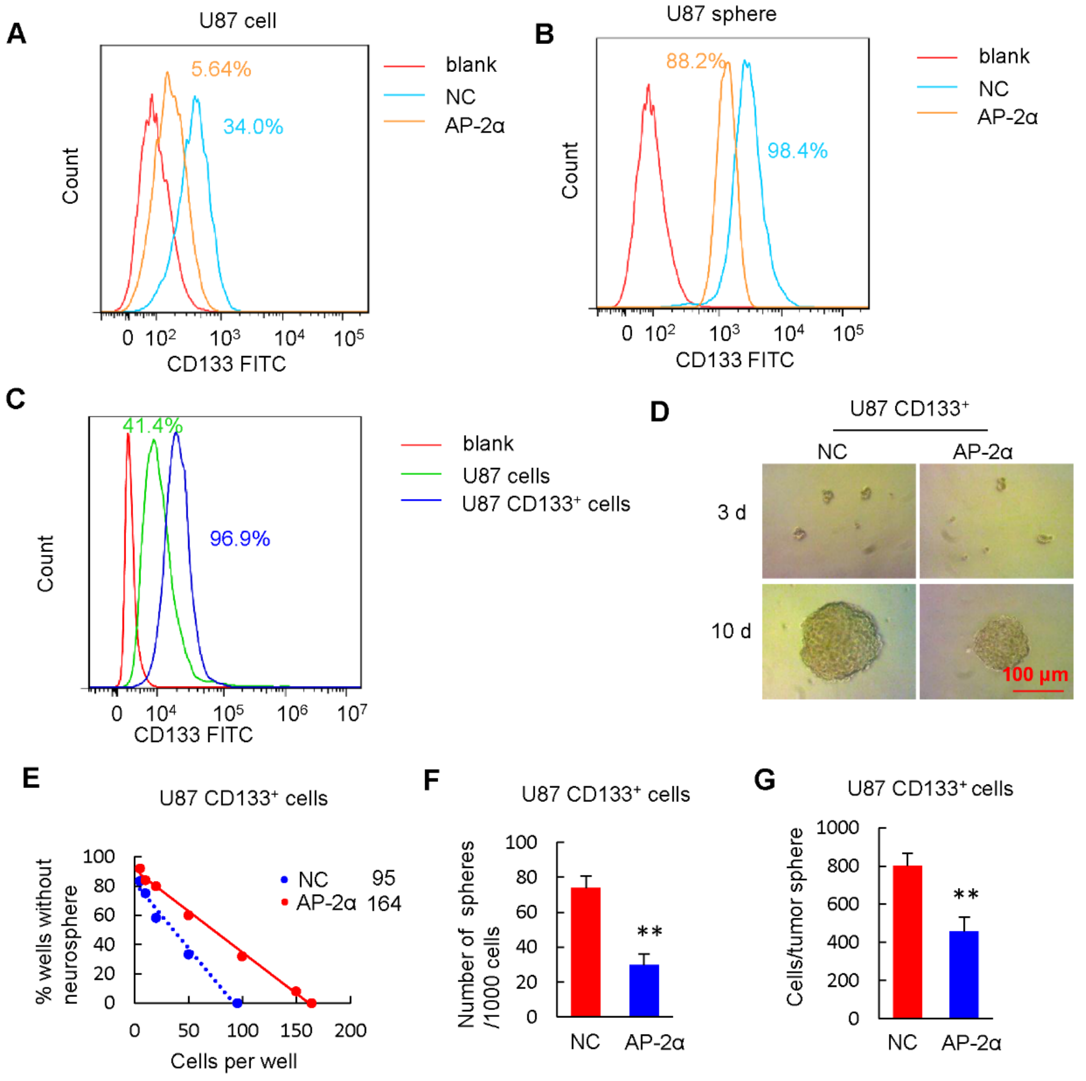

Figure S3. Effects of AP-2α overexpression on U87 CD133^{+} stem cells *in vitro*. (A) CD133 positive cells were detected in U87 cells by FACS analysis. The characteristic patterns of blank (anti-IgG FITC), NC and AP-2α groups (anti-CD133 FITC). (B) CD133 positive cells were detected using anti-CD133 FITC antibodies in U87 sphere cells by FACS analysis. (C) CD133 positive cells were sorted by magnetic beads and measured by FACS analysis. (D) Representative images of CD133^{+} U87 spheroid cells were shown. (E) A limiting dilution assay was performed to measure CD133^{+} tumor sphere formation. Cells were plated at 200, 100, 50, 20, 10, or 5 cells/well and cultured in stem cell-conditioned medium (n = 48 wells/condition, p =0.036). (F) Evaluation of the number of CD133^{+} spheres from 1000 cells on day 9. (G) Quantification of cell numbers per CD133^{+} U87 spheroid. **, p<0.01.

Figure S4

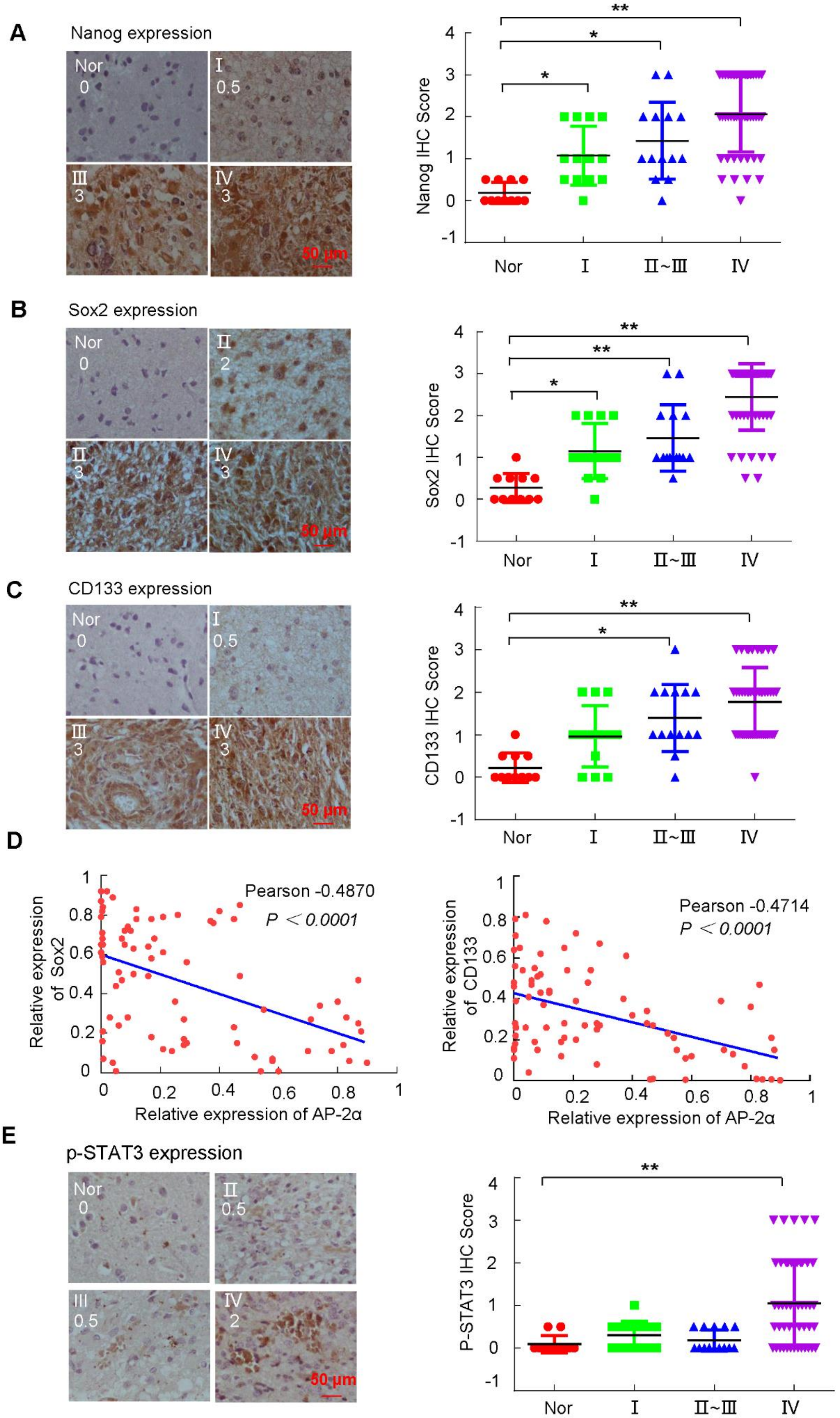
A
Nanog expression
Nor 0
I 0.5
Ⅲ 3
Ⅳ 3
50 μm
Nanog IHC Score
Nor
I
Ⅱ~Ⅲ
Ⅳ
B
Sox2 expression
Nor 0
Ⅱ 2
Ⅱ 3
Ⅳ 3
50 μm
Sox2 IHC Score
C
CD133 expression
Nor 0
I 0.5
Ⅲ 3
Ⅳ 3
50 μm
CD133 IHC Score
D
Pearson -0.4870
P < 0.0001
Relative expression of Sox2
Relative expression of AP-2α
Pearson -0.4714
P < 0.0001
Relative expression of CD133
Relative expression of AP-2α
E
p-STAT3 expression
Nor 0
Ⅱ 0.5
III 0.5
Ⅳ 2
50 μm
P-STAT3 IHC Score

Figure S4. The expression of AP-2α downstream genes in glioma tissues. Nanog expression (A), Sox2 expression (B) and CD133 expression (C) was examined by immunohistochemical analysis in 75 glioma and 11 adjacent normal tissues. Immunohistochemical scores of glioma and normal tissues stained with antibodies against anti-Nanog, anti-Sox2 and anti-CD133 were shown. (D) The correlation between AP-2α and Sox2 expression or AP-2α and CD133 expression was analyzed using GraphPad Prism. The staining intensity was scored with the proportion of positive cells. (E) The p-STAT3 expression was examined by immunohistochemical analysis in glioma tissues. Immunohistochemical scores of p-STAT3 staining were shown. *, $p<0.05$, **, $p<0.01$.
